# Metabolipidomic profiling reveals an age‐related deficiency of skeletal muscle pro‐resolving mediators that contributes to maladaptive tissue remodeling

**DOI:** 10.1111/acel.13393

**Published:** 2021-06-02

**Authors:** James F. Markworth, Lemuel A. Brown, Eunice Lim, Jesus A. Castor‐Macias, Jacqueline Larouche, Peter C. D. Macpherson, Carol Davis, Carlos A. Aguilar, Krishna Rao Maddipati, Susan V. Brooks

**Affiliations:** ^1^ Department of Molecular & Integrative Physiology University of Michigan Ann Arbor MI USA; ^2^ Department of Biomedical Engineering University of Michigan Ann Arbor MI USA; ^3^ Department of Pathology Lipidomics Core Facility Wayne State University Detroit MI USA

**Keywords:** aging, cellular immunology, inflammation, injury, mass spectrometry, sarcopenia, satellite stem cell, skeletal muscle

## Abstract

Specialized pro‐resolving mediators actively limit inflammation and support tissue regeneration, but their role in age‐related muscle dysfunction has not been explored. We profiled the mediator lipidome of aging muscle via liquid chromatography‐tandem mass spectrometry and tested whether treatment with the pro‐resolving mediator resolvin D1 (RvD1) could rejuvenate the regenerative ability of aged muscle. Aged mice displayed chronic muscle inflammation and this was associated with a basal deficiency of pro‐resolving mediators 8‐oxo‐RvD1, resolvin E3, and maresin 1, as well as many anti‐inflammatory cytochrome P450‐derived lipid epoxides. Following muscle injury, young and aged mice produced similar amounts of most pro‐inflammatory eicosanoid metabolites of cyclooxygenase (e.g., prostaglandin E_2_) and 12‐lipoxygenase (e.g., 12‐hydroxy‐eicosatetraenoic acid), but aged mice produced fewer markers of pro‐resolving mediators including the lipoxins (15‐hydroxy‐eicosatetraenoic acid), D‐resolvins/protectins (17‐hydroxy‐docosahexaenoic acid), E‐resolvins (18‐hydroxy‐eicosapentaenoic acid), and maresins (14‐hydroxy‐docosahexaenoic acid). Similar absences of downstream pro‐resolving mediators including lipoxin A_4_, resolvin D6, protectin D1/DX, and maresin 1 in aged muscle were associated with greater inflammation, impaired myofiber regeneration, and delayed recovery of strength. Daily intraperitoneal injection of RvD1 had minimal impact on intramuscular leukocyte infiltration and myofiber regeneration but suppressed inflammatory cytokine expression, limited fibrosis, and improved recovery of muscle function. We conclude that aging results in deficient local biosynthesis of specialized pro‐resolving mediators in muscle and that immunoresolvents may be attractive novel therapeutics for the treatment of muscular injuries and associated pain in the elderly, due to positive effects on recovery of muscle function without the negative side effects on tissue regeneration of non‐steroidal anti‐inflammatory drugs.

## Graphical abstract

Chronic low‐grade inflammation of aging muscle was associated with a basal deficiency of maresin 1, resolvin E3, 8‐oxo‐resolvin D1, and anti‐inflammatory fatty acid epoxides. Aged mice produced normal amounts of most prostaglandins following muscle injury but were deficient in local biosynthesis of markers of the lipoxins, E‐resolvins, D‐resolvins, and maresins. Systemic treatment with resolvin D1 suppressed inflammatory cytokine expression, limited muscle fibrosis, and improved functional recovery.

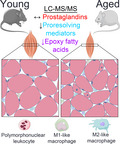

## 1 INTRODUCTION

Aging results in a progressive decline in skeletal muscle mass and function that contributes to frailty, loss of mobility, and increased mortality in the elderly (Faulkner et al., 2007). Aged muscles are also more susceptible to damage and have a reduced ability to regenerate successfully if injured (Blau et al., 2015). Potential sources of this dysfunction include a chronic accumulation of macrophages (MΦ) within muscle in advanced age (Wang et al., 2015), as well as dysregulated acute myeloid cell responses to muscle injury (Sloboda et al., 2018). On this basis, targeting the immune system has shown promise to rejuvenate the regenerative capacity of aged muscle and limit age‐associated muscle wasting (Tidball et al., 2021).

The immune response to muscle injury begins with rapid infiltration of neutrophils, followed by blood monocyte‐derived MΦ that initially exhibit a pro‐inflammatory phagocytic phenotype, but later act to support muscle regeneration (Arnold et al., 2007). In general, resolution of the acute inflammatory response is actively controlled by endogenous specialized pro‐resolving mediators that inhibit further recruitment of neutrophils, while stimulating regenerative MΦ functions (Chiang & Serhan, 2020). Docosahexaenoic acid (DHA) is the precursor to pro‐resolving mediators including the D‐series resolvins (Serhan et al., 2002), protectins (Mukherjee et al., 2004), and maresins (Serhan et al., 2009). In endogenous routes of their biosynthesis, 15‐lipoxygenase (15‐LOX) initially oxygenates DHA to form 17(S)‐hydroxy‐DHA (17‐HDoHE), a key intermediate metabolite in production of both the D‐series resolvins (e.g., RvD1) and protectins (e.g., PD1) (Hong et al., 2003). Alternatively, the 12‐LOX pathway produces 14(S)‐hydroxy‐DHA (14‐HDoHE), a key pathway marker of the maresins (e.g., MaR1) (Serhan et al., 2009). Additional important members of this family of metabolites include the arachidonic acid (ARA) derived lipoxins (e.g., LXA_4_) (Serhan et al., 1984) and eicosapentaenoic acid (EPA) derived E‐series resolvins (e.g., RvE1) that are generated via the initial formation of 15(S)‐hydroxy‐eicosatetraenoic acid (15‐HETE) and 18(R)‐hydroxy‐eicosapentaenoic acid (18‐HEPE), respectively (Arita et al., 2005; Serhan et al., 1984).

Specialized pro‐resolving mediators are produced in response to muscle injury (Giannakis et al., 2019; Markworth et al., 2020; Sansbury et al., 2020; Zhang et al., 2016) or physiological stress (Gangemi et al., 2003; Markworth et al., 2013; Vella et al., 2019; Zheng et al., 2019) implicating a role for these metabolites in muscle remodeling (Markworth et al., 2016). Current approaches to clinical management of soft tissue injuries focus predominantly on blocking cyclooxygenase (COX) mediated production of pro‐inflammatory prostaglandins with non‐steroidal anti‐inflammatory drugs (NSAIDs). However, NSAIDs also interfere with biosynthesis of pro‐resolving mediators (Markworth et al., 2013), which can delay timely resolution of inflammation (Schwab et al., 2007), with potential deleterious effects on muscle repair (Markworth et al., 2016). In contrast to NSAIDs, resolution agonists can stimulate muscle repair and may thus offer an attractive alternative to classical anti‐inflammatory approaches (Giannakis et al., 2019; Markworth et al., 2020; McArthur et al., 2020; Sansbury et al., 2020; Zhang et al., 2016).

Aging is associated with a deficiency of specialized pro‐resolving mediators in peritoneal fluid (Arnardottir et al., 2014), heart (Halade et al., 2016), central nervous system (Krashia et al., 2019), and lungs (Rymut et al., 2020). Indeed, mice lacking the key resolution sensor, lipoxin A_4_/formyl peptide receptor 2 (ALX/FPR2), develop a premature aging phenotype (Tourki et al., 2020). Conversely, treatment of aged mice with RvD1, an endogenous ALX/FPR2 ligand, can stimulate resolution in models of peritonitis (Arnardottir et al., 2014) and ischemia‐perfusion induced lung injury (Rymut et al., 2020). RvD1 was also recently shown to be of therapeutic benefit in a rat model of Parkinson's disease by modulating neuroinflammation (Krashia et al., 2019). The endogenous role and potential therapeutic applicability of specialized pro‐resolving mediators in the context of skeletal muscle aging have, however, not been examined.

In the current study, we investigated the effect of aging on the basal mediator lipidome of skeletal muscle as well as intramuscular lipid mediator production in response to sterile injury. Furthermore, we tested whether RvD1 treatment could limit inflammation and stimulate muscle regeneration in aged mice.

## 2 RESULTS

### 2.1 Age‐associated loss of muscle mass and strength

**FIGURE 1:**
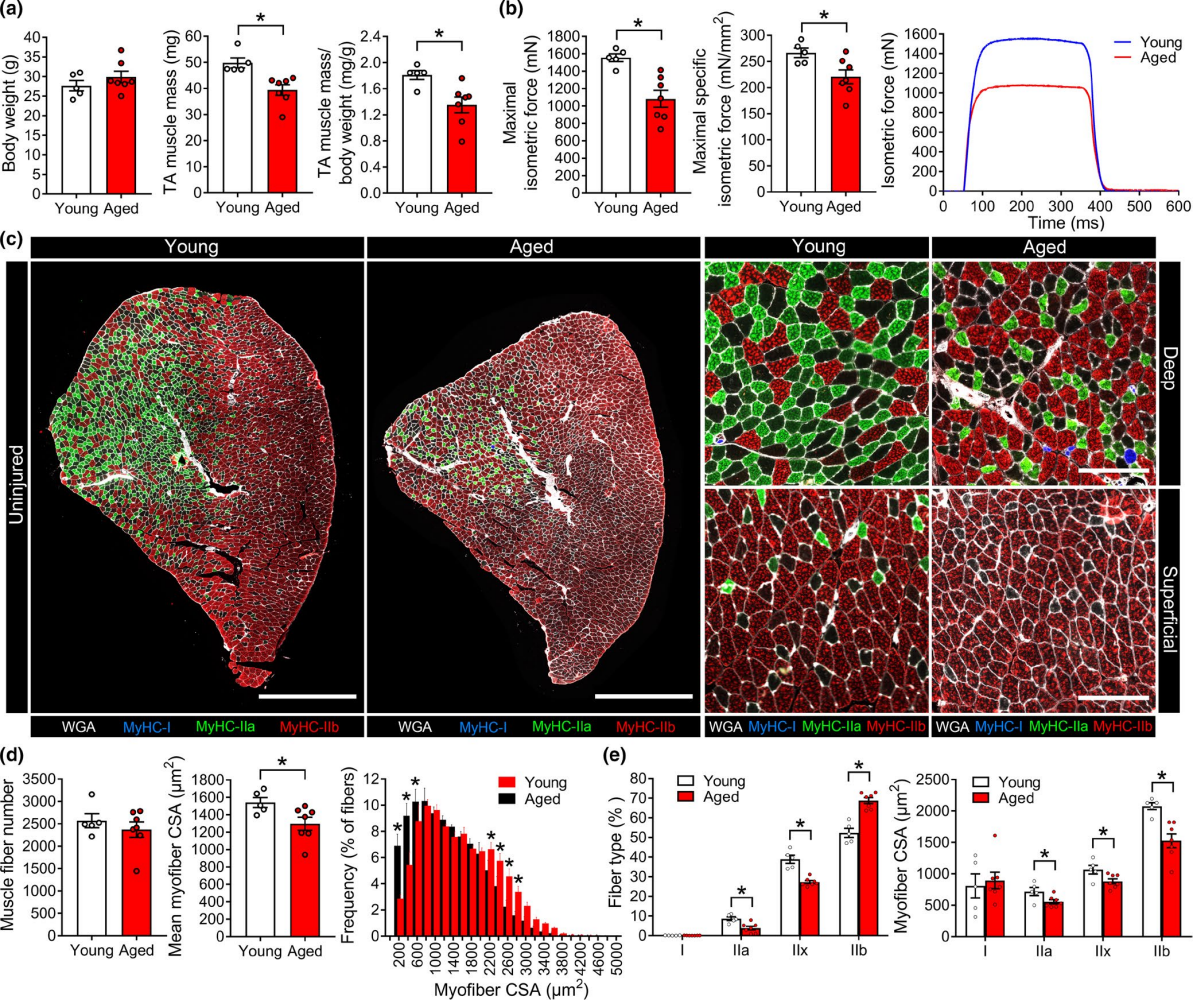
Age‐associated muscle wasting. (a) Bodyweight, tibialis anterior (TA) muscle mass, and relative TA mass of uninjured young and aged C57BL/6 mice. (b) Maximal nerve‐stimulated in‐situ isometric force (P_o_, mN) generated by young and aged TA muscles were measured and used to calculate maximal specific force (sP_o_, mN/mm^2^). (c) TA cross‐sections were stained with antibodies for type I, IIa, and IIb myosin heavy chain. Type IIx fibers remain unstained (black). The extracellular matrix was labeled with wheat germ agglutinin (WGA). Scale bars are 1000 µm. Right panels show representative fields of view from deep and superficial regions of the TA. Scale bars are 200 µm. The total number and cross‐sectional area (CSA) of each myofiber and its corresponding fiber type were determined using MuscleJ software. (d) Quantification of total myofiber number, mean fiber CSA, and fiber size‐frequency distribution. (e) Fiber type composition and mean fiber CSA split by fiber type. Bars show the mean ± SEM of 5–7 mice per group with dots representing data from each individual mouse. *Denotes *p* < 0.05 vs. young mice by two‐tailed unpaired *t*‐test

Aged mice (26–28 months) had similar bodyweights, but lower tibialis anterior (TA) muscle mass than young mice (4–6 months) (Figure 1a). Maximal in‐situ nerve‐stimulated TA isometric force (P_o_) was lower in aged mice and this deficit persisted after accounting for their smaller muscle size (specific P_o_, sP_o_) (Figure 1b). TA muscles from aged mice contained similar total numbers of myofibers but had a substantial reduction in mean myofiber cross‐sectional area (CSA) (Figure 1c, 1d). Aging also resulted in a loss of type IIa and IIx fibers, an increase in IIb fibers, and a reduction in mean CSA for all type II fibers (Figure 1c, 1e). These data show that these mice developed a robust sarcopenic phenotype in advanced age.

### 2.2 Chronic low‐grade inflammation of aged muscle is associated with a lack of anti‐inflammatory and pro‐resolving lipid mediators

**FIGURE 2:**
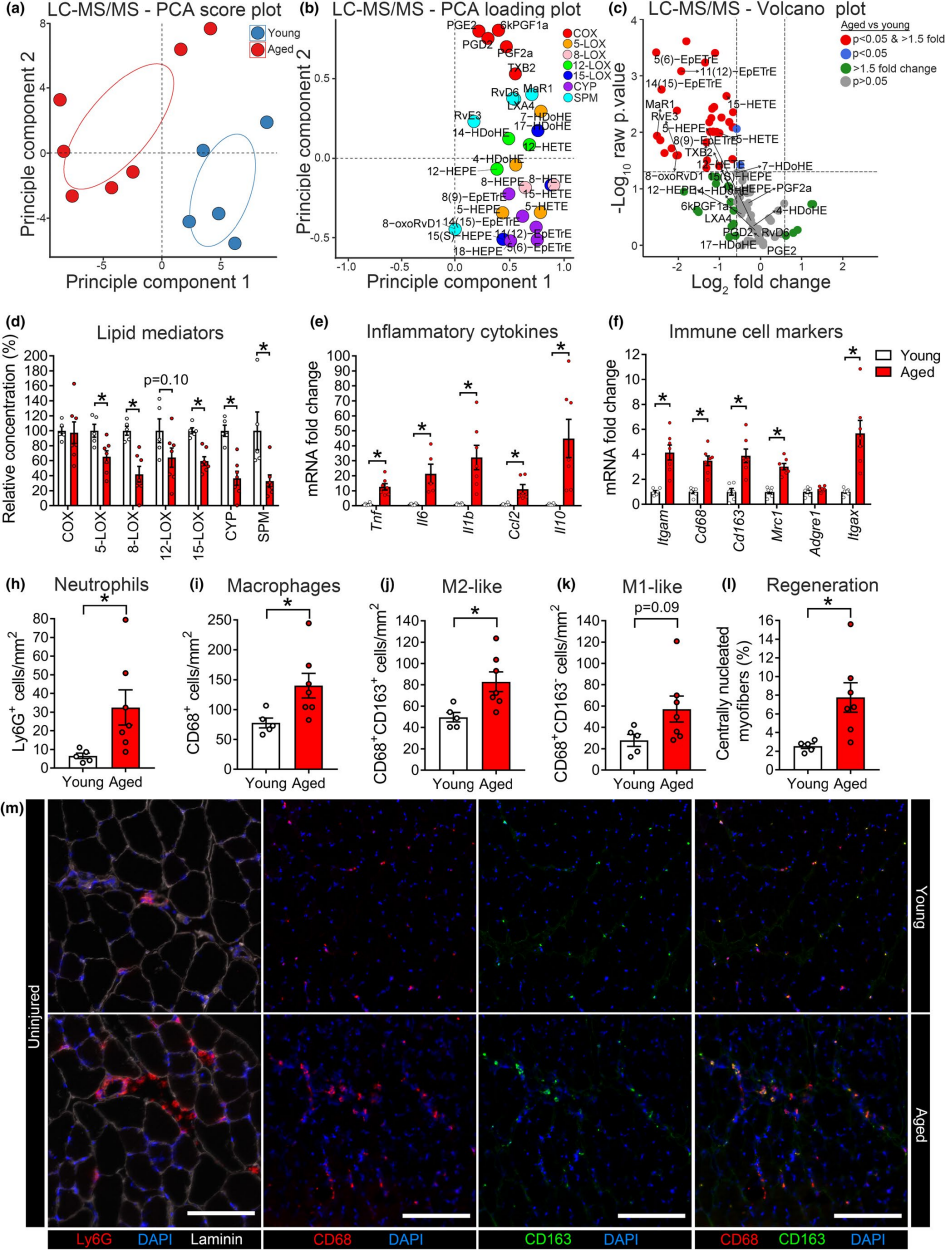
Chronic inflammation of aged muscle is associated with a deficiency of pro‐resolving lipid mediators. (a, b) Principle component analysis (PCA) score and loading plots of the mediator lipidome of uninjured TA muscles from young and aged C57BL/6 mice as determined by liquid chromatography‐tandem mass spectrometry (LC‐MS/MS). (c) Volcano plots summarizing the magnitude and statistical significance of difference between aged and young mice for each individual detected lipid mediator. Complete volcano plot data are shown in Table S2. (d) Relative pooled concentrations (pmol/mg tissue) of lipid mediator metabolites of ARA, EPA, and DHA derived from major enzymatic biosynthesis pathway in young and aged mice. Linoleic acid metabolites (e.g., HODEs and EpOMEs) are excluded and shown separately in Table S1. (e, f) TA mRNA expression of inflammation‐related genes as determined by real‐time quantitative reverse transcription PCR (RT‐qPCR). (h–l) Quantification of intramuscular number of neutrophils (Ly6G^+^ cells), total macrophages (MΦ) (CD68^+^ cells), M2‐like MΦ (CD68^+^CD163^+^ cells), M1‐like MΦ (CD68^+^CD163^−^ cells), and centrally nucleated (regenerating) myofibers. (m) Representative staining of cross‐sections of young and aged TA muscles. Scale bars are 100 µm for neutrophils (Ly6G panels) and 200 µm for MΦ (CD68 and CD163 panels). Bars show the mean ± SEM of 5–7 mice per group with dots representing data from each individual mouse. *Denotes *p* < 0.05 vs. vehicle group by two‐tailed unpaired *t*‐test

In order to investigate the mechanisms that may contribute to age‐associated muscle atrophy and weakness, we first performed targeted liquid chromatography‐tandem mass spectrometry‐based (LC‐MS/MS) based profiling of uninjured TA muscle homogenates from young and aged mice. Intramuscular concentrations of all detected metabolites are presented in Tables S1A–E. Unsupervised principle component analysis (PCA) score plots revealed a clear clustering of samples by age (Figure 2a). Corresponding loading plots show key representative metabolites from each major enzymatic pathway that contributed to this response (Figure 2b). Overall, the lipid mediator profile of young muscle was characterized by an abundance of polyunsaturated fatty acid (PUFA) metabolites, many of which are common products of the 5‐LOX, 15‐LOX, and CYP pathways. Of the 98 total lipid mediator species detected in muscle homogenates, 35 were present at significantly lower concentration (1.5‐fold, *p* < 0.05) in samples from aged mice, while none were significantly enriched (Figure 2c). A complete list of fold changes, *p*‐values, and associated false discovery rates for each analyte is shown in Table S2A.

**FIGURE 3:**
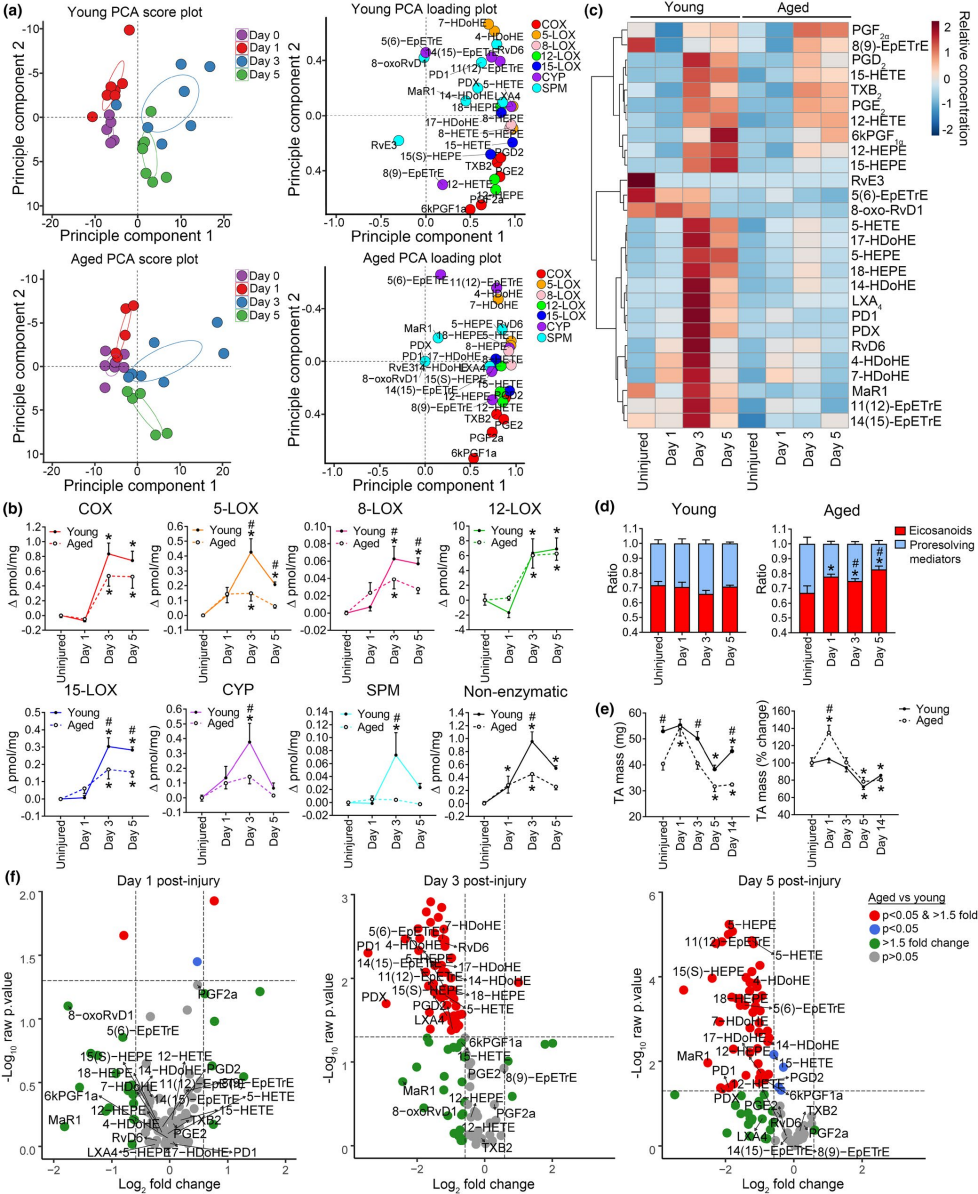
Impact of age on local shifts in lipid mediator biosynthesis following muscle injury. (a) Young and aged C57BL/6 mice received bilateral intramuscular injection of the TA muscle with 50 µL of 1.2% barium chloride (BaCl_2_) to induce myofiber injury. TA muscles were collected at 1, 3, and 5 days’ post‐injury for analysis of intramuscular lipid mediator concentrations via LC‐MS/MS. PCA score and loading plots show global shifts in the mediator lipidome of injured muscle. (b) Changes in pooled lipid mediator metabolites of ARA, EPA, and DHA from potential biosynthetic routes. Data are presented as the change in concentration (pmol/mg tissue) from age‐matched uninjured muscles displayed in Figure 2D. Linoleic acid metabolites (e.g., HODEs and EpOMEs) are excluded and shown separately in Table S1. (c) Heat map summarizing average temporal shifts in representative individual lipid mediators. Statistical analysis of these analytes by two‐way ANOVA is shown in Figure S2. (d) Shifts in the balance of major eicosanoids (TXB_2_, PGD_2_, PGE_2_, PGF_2α_, 6kPGF_1α_, and 5‐, 12‐, 15‐HETEs) relative to detected pro‐resolving mediators (RvE3, 8‐oxoRvD1, LXA_4_, RvD6, PD1, PDX, and MaR1) and related pathway markers (5‐, 18‐HEPEs and 4‐, 7‐, 14, 17‐ HDoHEs) following muscle injury. (e) Changes in the mass of muscle samples used for lipidomic profiling. (f) Volcano plots summarizing the magnitude and statistical significance of difference between aged and young mice for each individual detected lipid mediator. Bars show the mean ± SEM of 5–7 mice per group. (b–e) *denotes *p* < 0.05 change from age‐matched uninjured muscles as determined by two‐way ANOVA with Holm‐Sidak post hoc tests. #denotes *p* < 0.05 between young and aged mice at a given time‐point by two‐way ANOVA with Holm‐Sidak post hoc tests

Cumulative metabolites of the COX‐1 and 2 pathways encompassing the thromboxanes and prostaglandins were similarly abundant in young and aged muscle (Figure 2d, Table S1A) with no individual COX metabolites differing between young and aged mice (Table S2A). In contrast, pooled products of the 5‐LOX and 15‐LOX pathways were substantially lower in aged mice (Figure 2d, Table S1B). Most notably, this included 15‐HETE, the primary 15‐LOX‐derived metabolites of ARA which is a key pathway marker of the lipoxins (Tables S1B and S2A). On the other hand, 17‐HDoHE, the primary 15‐LOX metabolite of DHA and key D‐series resolvin biosynthetic marker did not differ between young and aged muscle at baseline. Nevertheless, 5‐LOX metabolites of both ARA [5‐hydroxy‐eicosatetraenoic acid (5‐HETE)] and DHA [7‐hydroxy‐docosahexaenoic acid (7‐HDoHE)], additional pathway markers of the lipoxins and D‐resolvins, respectively, were lower in aged versus young muscle (Tables S1B and S2A). Finally, the overall activity of the CYP pathway, which primarily generates anti‐inflammatory epoxide metabolites such as the epoxy‐eicosatrienoic acids (EpETrEs), but also produces the key E‐series resolvin pathway marker 18‐HEPE, was markedly lower in aged muscle (Figure 2d, Table S1C). Indeed, 18‐HEPE was ~30% lower in aged versus young muscle, although this did not reach statistical significance (*p* = 0.14) (Table S2A). Downstream di‐ and trihydroxy‐PUFA metabolites, which are produced endogenously by the sequential action of two or more different LOX and/or CYP isoforms, were generally present at very low concentrations in muscle and were often below the limits of detection (Table S1D). Nevertheless, bioactive specialized pro‐resolving mediators (abbreviated as SPM in Figures 2 and 3) including LXA_4_, resolvin D6 (RvD6), MaR1, and MaR1_n‐3 DPA_ were detected in all samples, while resolvin E3 (RvE3) and 8‐oxo‐RvD1 were detected only in young muscle (Figure 2c, Table S1D). There was an overall basal deficiency of pooled specialized pro‐resolving mediators in aged mice (Figure 2d), predominantly attributable to significantly lower concentrations of MaR1 (Table S1D and S2A).

The deregulated mediator lipidome of aged muscle was accompanied by increased muscle mRNA expression of cytokines and immune cell markers, as well as greater intramuscular numbers of neutrophils, total MΦ, M2‐like MΦ, and to a lesser extent M1‐like MΦ (*p* = 0.09) (Figure 2h–k), although the ratio of M2:M1‐like MΦ did not differ between young and aged mice (2.17 vs. 1.64, *p* = 0.34). This chronic accumulation of MΦ in aged muscle was further confirmed by flow cytometry (Figure S1). Inflammation of aged muscle was associated with increased centrally nucleated fibers supporting ongoing myofiber degeneration/regeneration (Figure 2l). Representative images illustrating intramuscular myeloid cell populations are shown in Figure 2m. Overall, the data show that chronic inflammation of aging muscle is characterized by a basal deficiency of pro‐resolving lipid mediators.

### 2.3 Aged mice mount a deficient specialized pro‐resolving lipid mediator response to muscle injury

We next assessed the effect of aging on local lipid mediator biosynthesis following muscle injury induced by intramuscular injection of barium chloride (BaCl_2_). There was a minor shift in global muscle lipid mediator profile at day 1 post‐injury (Figure 3a), due predominantly to a rapid increase in likely non‐enzymatic PUFA metabolites (Figure 3b, Table S1E). This was followed by a marked shift in muscle lipid mediator profile at day 3 post‐injury attributable to increased concentrations of many major products of the COX, LOX, and CYP pathways (Figure 3a,b, Table S1A–D).

When compared to young mice, aged mice mounted an overall deregulated lipid mediator response as evidenced by less separation of clusters of samples obtained at distinct time‐points within respective PCA score plots (Figure 3a). Analysis of intramuscular lipid mediators pooled over major biosynthetic pathways by two‐way ANOVA revealed that aged and young mice produced similar cumulative amounts of COX, 12‐LOX, and CYP products following injury (age ×time effects of *p* = 0.33, *p* = 0.74, *p* = 0.27, respectively) (Figure 3b). In contrast, aged mice showed diminished local production of cumulative metabolites of the 5‐, 8‐, and 15‐LOX pathways, as well as a marked deficiency in pooled detected downstream bioactive specialized pro‐resolving mediators (age ×time *p* = 0.018, *p* = 0.048, *p* = 0.013, *p* = 0.036, respectively) (Figure 3b).

A heat map summarizing the temporal shifts in key individual lipid mediator species is shown in Figure 3c. There were age‐related deficiencies in biosynthesis of LXA_4_, RvD6, protectin D1 (PD1), protectin DX (PDX), and MaR1 (Tables S1D and S2B–D). Key pathway markers of the lipoxins (15‐HETE), D‐resolvins (17‐HDoHE), E‐resolvins (18‐HEPE), and maresins (14‐HDoHE) were also all produced to a significantly lesser extent in aged muscle following injury (Tables S1B and S2C,D). On the other hand, young and aged mice displayed largely equivalent production of many pro‐inflammatory eicosanoid metabolites of the COX pathway [e.g., thromboxane B_2_ (TXB_2_), prostaglandin E_2_ (PGE_2_)_,_ and prostaglandin F_2α_ (PGF_2α_)] and 12‐LOX pathway [e.g., 12‐hydroxy‐eicosatetraenoic acid (12‐HETE)] (Tables S1A and S2B–D). Notable exceptions were prostaglandin D_2_ (PGD_2_) and 13,14‐dihydro‐15‐keto PGD_2_, the cyclopentenone prostaglandins J_2_ (PGJ_2_) and 15‐deoxy‐^Δ^12,14‐ PGJ_2_, as well as prostaglandin I_2_ (PGI_2_ or prostacyclin), measured as its degradation product 6‐keto‐prostaglandin F_1α_ (6kPGF_1α_), all of which were significantly lacking in aged muscle following injury. The blunted pro‐resolving mediator response in aged muscle resulted in a relative overabundance of pooled eicosanoids following injury (Figure 3d). Kinetic data for major individual prostaglandins detected specialized pro‐resolving mediators, and related LOX and CYP derived pathway markers are shown in Figure S2.

With the exception of an initial transient increase in muscle mass at day 1 post‐injury in aged mice, temporal shifts in the masses of the TA muscles used for lipidomic profiling were similar between young and aged mice (Figure 3e). Volcano plots summarizing the overall differences between aged and young muscle mediator lipidomes for all detected analytes at each time‐point are shown in Figure 3f and Table S2B–D. These findings demonstrate that aging results in a marked imbalance in local biosynthesis of inflammatory and resolving lipid mediators following muscle damage.

### 2.4 Resolvin D1 treatment suppresses intramuscular inflammatory cytokines but does not limit excessive leukocyte infiltration of aged muscle

Since aging was associated with impaired pro‐resolving mediator biosynthesis in response to muscle injury, we aimed to investigate whether treatment of aged mice with exogenous pro‐resolving molecules might limit muscle inflammation and improve regeneration. We chose to test RvD1 due to its well‐established dose‐response pharmacokinetics (Sun et al., 2007), stimulatory effects on muscle regeneration in young mice (Markworth et al., 2020; Sansbury et al., 2020), and ability to protect aged mice from ischemia‐reperfusion induced lung injury (Rymut et al., 2020). Finally, 17‐HDoHE, the primary 15‐LOX‐derived precursor to RvD1 was detected in muscle, markedly increased following injury, but significantly impaired in aged mice.

We first confirmed that RvD1 treatment stimulated phagocytosis by bone marrow‐derived MΦ in‐vitro and showed that MΦ from aged mice maintained the intrinsic ability to respond effectively to RvD1 (Figure S3). We then treated aged mice with RvD1 at a dose of 100 ng/day administered by intraperitoneal injection. This route and dose were based on many prior studies (e.g., Markworth et al., 2020; Sansbury et al., 2020). We did not measure its pharmacokinetics, but RvD1 is well‐known to rapidly appear in peripheral circulation following intraperitoneal injection (Krashia et al., 2019).

**FIGURE 4:**
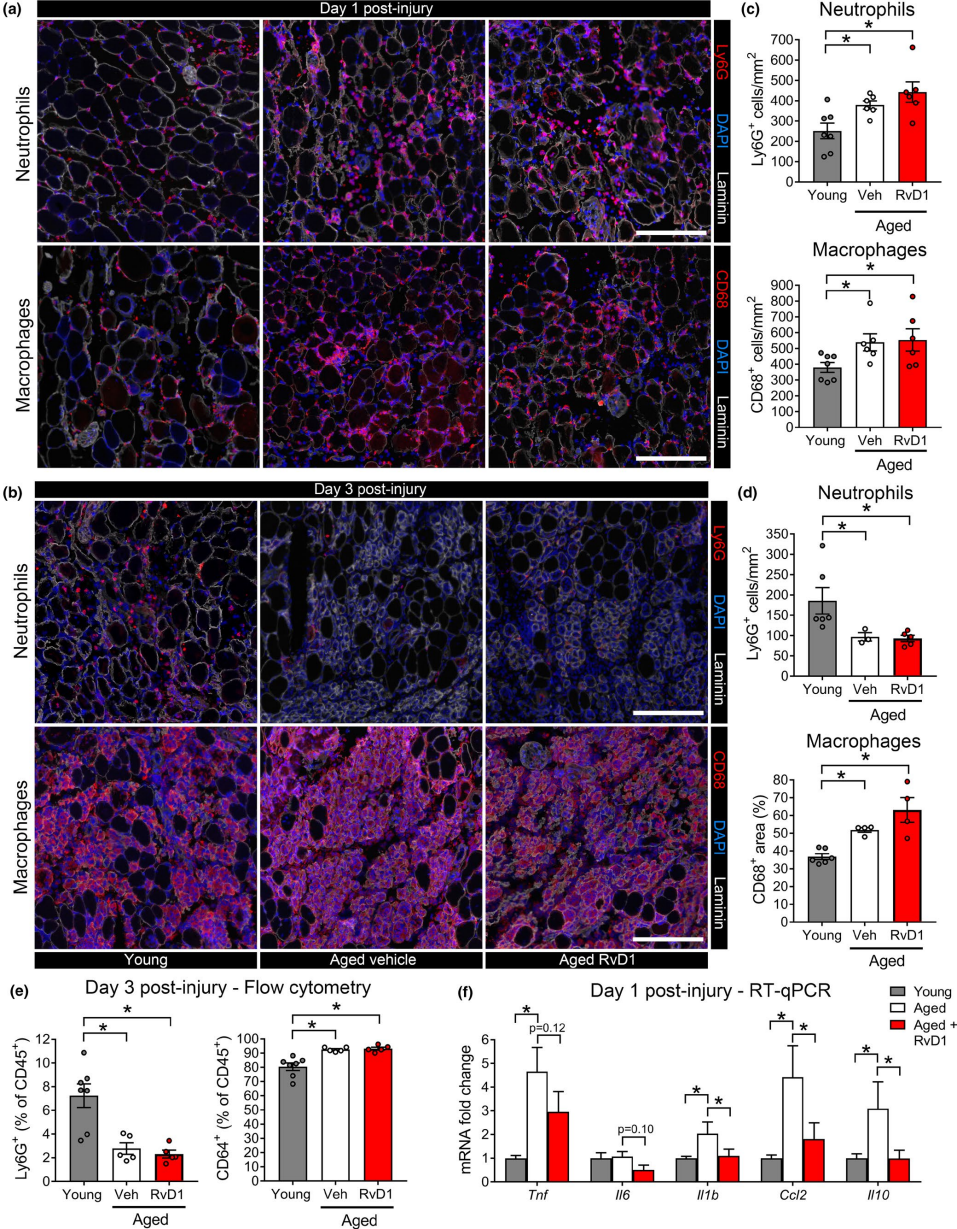
The effect of aging and RvD1 treatment on the inflammatory response to muscle injury. (a, b) Young and aged C57BL/6 mice received bilateral intramuscular injection of the TA muscle with 50 µl of 1.2% BaCl_2_ to induce myofiber injury. Aged mice were treated daily with RvD1 (100 ng) or vehicle (0.1% ethanol) via intraperitoneal (IP) injection. TA muscles were collected at day 1 and 3 post‐injury and muscle cross‐sections were stained for neutrophils (Ly6G) or monocytes/macrophages (MΦ, CD68). Cell nuclei and the basal lamina were counterstained with DAPI and a laminin antibody respectively. Scale bars are 200 µm. (c, d), Quantification of neutrophils (Ly6G^+^ cells) and MΦ (CD68^+^ cells) in injured muscle at day 1 and 3 post‐injury. (e) Intramuscular neutrophils (Ly6G^+^ cells) and MΦ (CD64^+^ cells) as percentage of total intramuscular leukocytes (CD45^+^ cells) as determined by flow cytometry. (f) Quantification of mRNA expression of muscle cytokine expression at day 1 post‐injury by RT‐PCR. Bars show the mean ± SEM of 4–7 mice per group with dots representing data from each individual mouse. *Denotes *p* < 0.05 between groups by one‐way ANOVA with pairwise LSD post hoc tests

At day 1 post‐injury, there was a large increase for both age groups in intramuscular neutrophils (Ly6G^+^ cells) and MΦ (CD68^+^ cells) that was followed by a subsequent decline in Ly6G^+^ cells and progressive MΦ recruitment peaking at day 3 (Figure 4a,b). When compared to young mice, aged mice initially showed relatively greater recruitment of Ly6G^+^ cells at 1‐day post‐injury, but more significant clearance by day 3 (Figure 4c,d), with a relatively greater intramuscular MΦ presence at both day 1 and day 3 post‐injury (Figure 4c,d). Treatment of aged mice with RvD1 did not influence myeloid cell numbers in muscle (Figure 4c,d).

To confirm the surprising finding that despite a markedly deficient local biosynthetic response for specific pro‐resolving mediators in aged mice, the rate of clearance of neutrophils from injured muscle was accelerated, we repeated these experiments for analysis of the whole intramuscular single cell population by flow cytometry. At day 3 post‐injury ~8% of intramuscular leukocytes (CD45^+^ cells) were Ly6G^+^ in young mice, compared with only ~2% in aged mice (Figure 4e). This was accompanied by a parallel increase in the proportion of MΦ (CD64 ^+^ cells) in aged muscle. Treatment of aged mice with RvD1 did not influence the proportion of CD45^+^ cells that were either Ly6G^+^ or CD64^+^ (Figure 4e). RvD1 treatment did, however, reduce muscle mRNA expression of cytokines within the injured muscle (Figure 4f). Thus, RvD1 may still influence the inflammatory profile of intramuscular leukocytes despite not impacting their number.

### 2.5 Defective myofiber regeneration in aged mice is not influenced by RvD1

**FIGURE 5:**
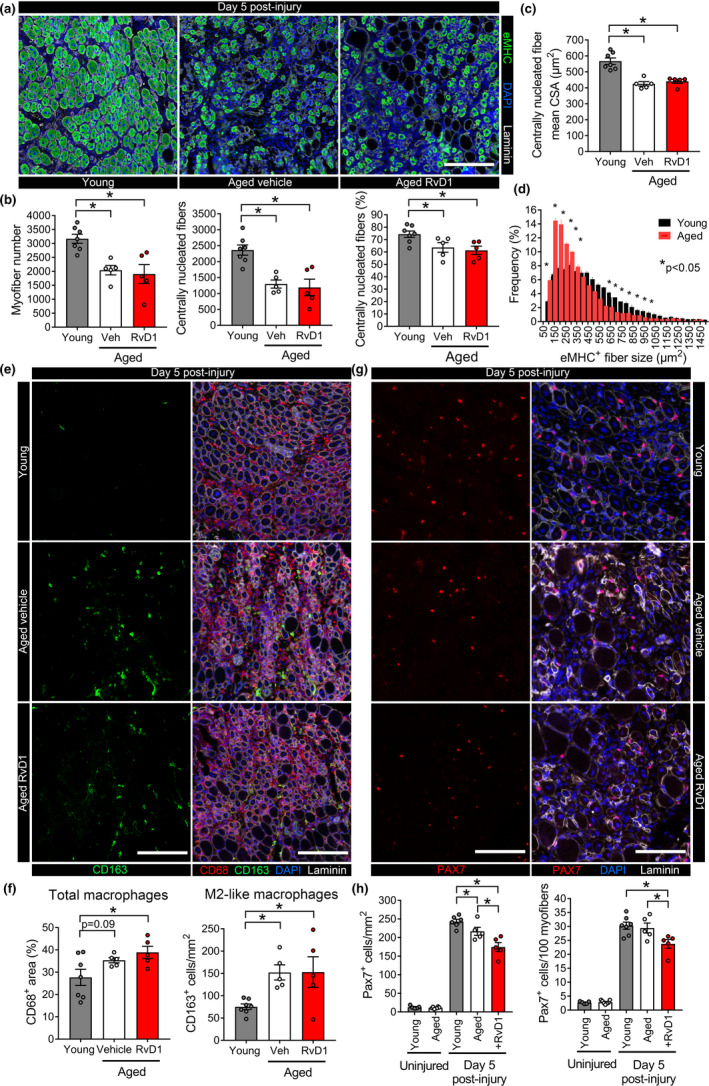
The effect of age and RvD1 treatment on myofiber regeneration. (a) Young and aged C57BL/6 mice received bilateral intramuscular injection of the TA muscle with 50 µl of 1.2% BaCl_2_ to induce myofiber injury. Aged mice were treated daily with RvD1 (100 ng) or vehicle (0.1% ethanol) by IP injection. TA muscles were collected at day 5 post‐injury and muscle cross‐sections were stained for embryonic myosin heavy chain (eMHC). Cell nuclei and the basal lamina were counterstained with DAPI and a laminin antibody respectively. Scale bars are 200 µm. (b) Quantitative of total myofiber number, regenerating (centrally nucleated) myofiber number, and the percentage of total myofibers undergoing regeneration as determined by MuscleJ. (c) Mean CSA of the regenerating myofiber population. (d) Frequency distribution regenerating myofiber CSA in young and aged mice. (e) Muscle cross‐sections were stained for total MΦ (CD68) and M2‐like MΦ (CD163). Scale bars are 200 µm. (f) Quantification of total MΦ infiltration as percentage of tissue area containing CD68^+^ staining and M2‐like MΦ (CD163^+^ cell) counts. (g) Muscle cross‐sections were stained for the muscle satellite cell marker Pax7 at day 5 post‐injury. TA muscles from age‐matched uninjured mice served as controls. Scale bars are 100 µm. (h) Quantification of satellite cell number expressed relative to tissue area or myofiber number. Bars are mean ± SEM of 5–7 mice per group with dots representing data for each individual mouse. *p < 0.05 by one‐way ANOVA with pairwise LSD post hoc tests for panels A–F or pairwise Holm‐Sidak post hoc tests for panel H

Many small myofibers with characteristic centrally located nuclei and robust expression of embryonic myosin heavy chain (eMHC) appeared by day 5 post‐injury in both young and aged mice (Figure 5a). When compared to young mice, regenerating muscle cross‐sections from aged mice contained fewer newly formed myofibers (as assessed by the combination of eMHC expression and associated centrally located myonuclei) (Figure 5b). In addition to a lack of fibers undergoing regeneration in aged mice, the myofibers that were regenerating were smaller in size (Figure 5c,d). RvD1 treatment did not influence regenerating fiber number or size (Figure 5a–c).

When compared to young mice, aged muscles tended to have more total MΦ at day 5 post‐injury (*p* = 0.09), and this was especially true for M2‐like MΦ (CD68^+^CD163^+^ cells) (Figure 5e). Treatment of aged mice with RvD1 did not impact intramuscular MΦ (Figure 5f). Muscle satellite cells (MuSC) increased markedly at day 5 post‐injury, aged mice showed lower numbers of MuSCs than young mice, and this was reduced even further by RvD1 treatment (Figure 5g,h). Thus, RvD1 treatment had a marked impact on the MuSC response to muscle damage but this did not translate to a clear positive or negative impact on myofiber regeneration.

### 2.6 RvD1 limits maladaptive remodeling of aged muscle and improves recovery of muscle function

**FIGURE 6:**
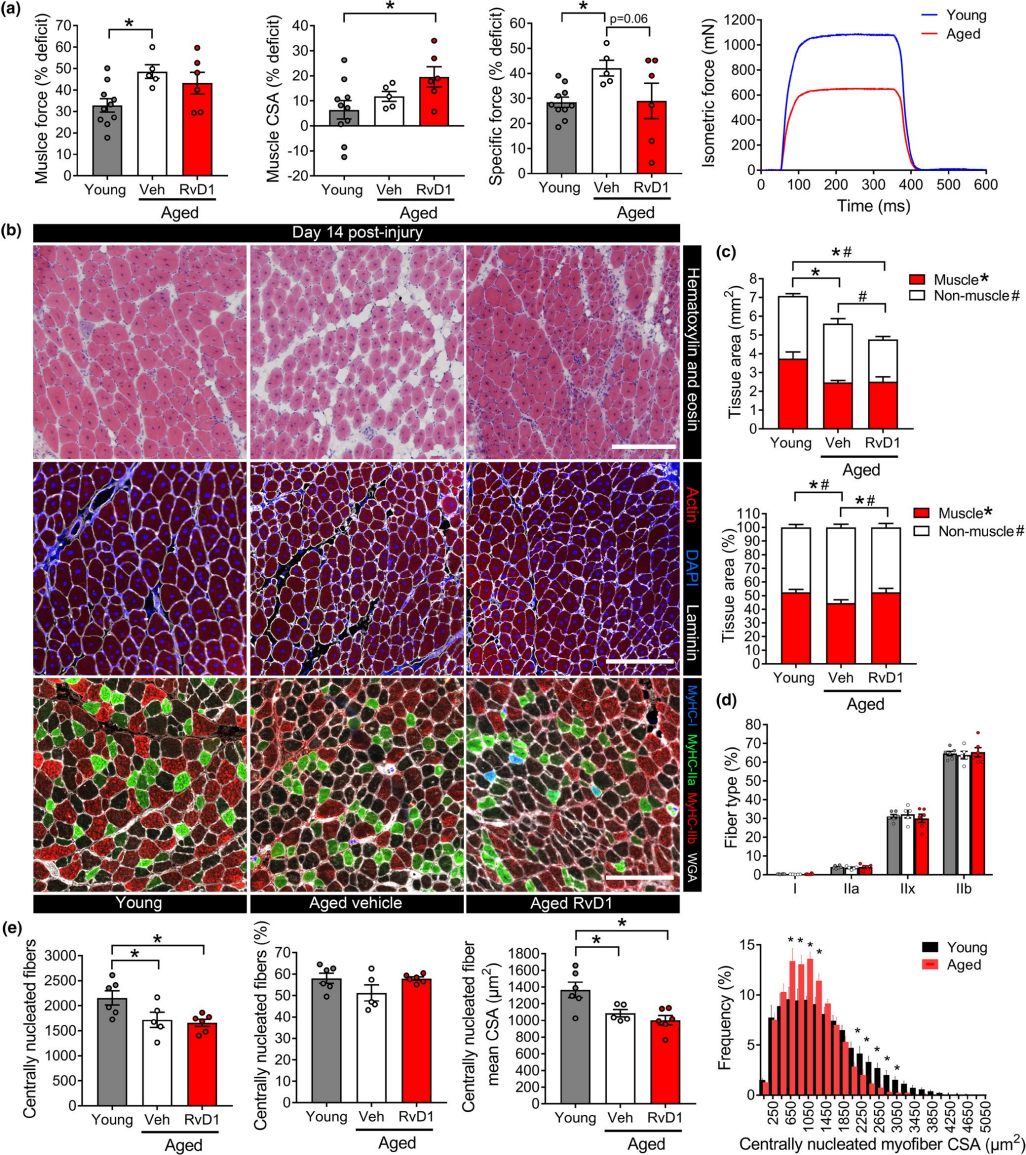
RvD1 limits maladaptive remodeling following muscle injury in aged mice. (a) Young and aged C57BL/6 mice received bilateral intramuscular injection of the TA muscle with 50 µl of 1.2% BaCl_2_ to induce myofiber injury. Aged mice were treated with daily IP injections of RvD1 (100 ng) or vehicle (0.1% ethanol) for 14 days. On day 14 post‐injury maximal nerve‐stimulated in‐situ isometric force (P_o_, mN) generated by the TA muscle was measured and used to calculate specific force (sP_o_, mN/mm^2^). Data are presented as percentage force deficit relative to age‐matched uninjured TA muscles shown in Figure 1B. (b) TA cross‐sections were stained for hematoxylin & eosin, conjugated phalloidin (actin), or with antibodies against type I, IIa, and IIb myosin heavy chain. Type IIx fibers remain unstained (black). Cell nuclei and the basal lamina were counterstained with DAPI and a laminin antibody on phalloidin slides. The extracellular matrix was stained with wheat germ agglutinin (WGA) to delineate myofiber boundaries on fiber type slides. Scale bars are 200 µm. Image analysis was performed using MuscleJ software. (c) Whole regenerating muscle CSA and relative amounts of tissue area containing contractile myofibers (actin^+^ area) compared with extracellular matrix (actin^−^ tissue area). (d) The fiber type composition of regenerating muscles from young and aged mice. (e) Quantification of regenerating (centrally nucleated) myofiber number, percentage of total fibers undergoing regeneration, and the mean CSA/frequency distribution of the centrally nucleated fiber population. Bars show mean ± SEM of 5–10 mice per group with dots representing data from each individual mouse *Denotes *p* < 0.05 by one‐way ANOVA with pairwise LSD post‐hoc tests

When expressed relative to age‐matched uninjured muscles (Figure 1), aged mice had greater deficits than young mice at 14 days post‐injury for P_o_ and much of this difference persisted after accounting for the smaller regenerating muscle size in aged mice (sP_o_), while treatment with RvD1 tended to improve this sPo deficit (*p* = 0.06) (Figure 6a). To investigate the basis for the functional improvement, we performed hematoxylin and eosin staining and found that compared to young mice, regenerating muscles from untreated aged mice had a greatly expanded interstitial space between their smaller regenerating myofibers (Figure 6b). Aged TA muscle cross‐sections were overall smaller in size than young mice and this was mainly due to a reduction in muscle fiber (actin^+^) area, while the amount of extracellular matrix (actin^−^ area) was similar to young muscle (Figure 6c). Because of this, regenerating aged muscles were comprised of a greater proportion of non‐contractile tissue, indicative of poorer muscle quality and reflected in the sP_o_ deficit. When compared to aged mice receiving vehicle treatment, RvD1 did not influence overall muscle size or total myofiber (actin^+^) area but did reduce the amount of intramuscular non‐contractile (actin^−^) tissue (Figure 6c). Consequently, the relative proportion of muscle that was comprised of functional myofibers was increased in aged mice treated with RvD1 when compared to untreated aged mice.

Neither age nor RvD1 treatment had a significant impact on the fiber type composition of regenerating muscles (Figure 6d) and both young and aged TA muscles were predominantly composed of regenerating myofibers at day 14 post‐injury (Figure 6e). When compared to young mice, aged muscles contained fewer regenerating myofibers which were on average smaller in size, but this was not influenced by RvD1 treatment. Aged muscles also still contained many more intramuscular MΦ than young mice even at 2‐weeks following muscle damage and treatment of aged mice with RvD1 did not influence this response (Figure S4).

## 3 DISCUSSION

We examined the effect of aging on the local lipid mediator response to muscle injury and tested whether systemic RvD1 treatment limits inflammation and improves muscle regeneration in aged mice. Aged mice produced similar amounts of pro‐inflammatory prostaglandins (e.g., PGE_2_) as young mice following injury, but displayed deficient intramuscular markers of specialized pro‐resolving mediator biosynthesis, as well as a lack of several downstream lipoxins, resolvins, protectins, and maresins. This deregulated lipid mediator response was associated with excessive inflammation, deficient myofiber regeneration, increased fibrosis, and delayed functional recovery. Treatment of aged mice with RvD1 limited inflammatory cytokine expression but did not impact myeloid cell recruitment or myofiber regeneration. Nevertheless, RvD1 limited maladaptive tissue remodeling resulting in improved recovery of muscle‐specific force. These findings reveal an age‐associated imbalance of intramuscular inflammatory and resolving lipid mediators and support immunoresolvents as an attractive alternative for the clinical management of muscle injuries and associated pain in the elderly, due to positive effects on recovery of strength without negative side effects of NSAIDs on muscle regeneration.

Consistent with recent reports, we found a chronic age‐associated increase in muscle neutrophils (Kawanishi & Machida, 2021; Li et al., 2020; Sloboda et al., 2018) and M2‐like MΦ (Cui et al., 2019; Jensen et al., 2020; Reidy et al., 2019; Sorensen et al., 2019; Wang et al., 2015). Aged mice also mounted a heightened acute inflammatory response to muscle injury, which is also consistent with prior studies (Blanc et al., 2020; Patsalos et al., 2018; Rahman et al., 2020; Sloboda et al., 2018). LC‐MS/MS‐based profiling revealed that this greater inflammation was associated with a deficiency in local biosynthesis of key pathway markers in the biosynthesis of the lipoxins (15‐HETE), E‐resolvins (18‐HEPE), D‐resolvins/protectins (17‐HDoHE), and maresins (14‐HDoHE). While di‐ and tri‐hydroxylated PUFA metabolites were only sporadically detectable within muscle homogenates, several downstream bioactive specialized pro‐resolving mediators including LXA_4_, RvD6, PD1, PDX, and MaR1 were also relatively lacking in aged muscle during regeneration.

Systemic treatment of aged mice with RvD1 has previously shown promise limiting leukocyte‐induced lung injury (Rymut et al., 2020) and modulating neuroinflammation in a rat model of Parkinson's disease (Krashia et al., 2019). Furthermore, we and other groups recently showed that RvD1 treatment improved regenerative outcomes following muscle injury in young mice (Markworth et al., 2020; Sansbury et al., 2020). In the current study, treatment with RvD1 suppressed both pro and anti‐inflammatory cytokines in muscle of aged mice, but in contrast to our prior study in young mice, did not reduce early MΦ infiltration (Markworth et al., 2020). Other inflammation‐related genes (e.g., TNFα, IL‐6) that were markedly suppressed post‐injury in young mice treated with RvD1 (Markworth et al., 2020), also only trended toward suppression in aged muscle here. Thus, RvD1 was unable to fully overcome heightened muscle myeloid cell infiltration in aged mice. This may relate to the increased inflammation‐related gene expression in aged muscle even before injury. Therefore, future studies should examine whether longer‐term immunoresolvent treatments may reduce chronic basal inflammation of aged muscle. Furthermore, a single immunoresolvent class may be insufficient for the overall resolution of inflammation following muscle injury, especially in aged mice which were deficient in markers of all major pro‐resolving mediators. Therefore, therapeutic administration of different specialized pro‐resolving mediators and/or combinatorial treatments may have additional therapeutic effects on age‐related muscle dysfunction.

The 12‐LOX pathway is classically known for producing the pro‐inflammatory eicosanoid 12‐HETE. However, the maresin family of specialized pro‐resolving mediators is also formed via the 12‐LOX pathway, by the initial conversion of DHA to 14‐HDoHE (Serhan et al., 2009). Both 12‐HETE and 14‐HDoHE increased in response to muscle injury in the current study and while the 14‐HDoHE response was markedly blunted in aged compared with young mice, 12‐HETE was not impacted by aging. How two distinct metabolic products of the same enzymatic pathway can be differently modulated in aging muscle remains unclear, but several plausible mechanisms exist. Firstly, the deficient 14‐HDoHE response might originate from a deficiency in DHA, rather than defects in 12‐LOX activity. Secondly, multiple 12‐LOX isoforms exist, which differ in their substrate specificity, enzyme kinetics, and cell‐type expression profiles and only specific 12‐LOX isoforms may be deregulated in aged muscle. Finally, production of specialized pro‐resolving mediators involves multiple steps, often via transcellular biosynthetic routes involving two or more enzymes. Therefore, the lack of maresins in aged muscle might also result from 12‐LOX independent mechanisms entirely, although our 14‐HDoHE data would argue against this conclusion.

We also observed a marked deficit in aged muscle prior to injury in many epoxide metabolites derived from the less well‐explored CYP pathway which possess anti‐inflammatory actions (Christmas, 2015). A recent study showed that PUFA epoxides also stimulate the resolution of inflammation and could thus be classed as pro‐resolving mediators themselves (Gilroy et al., 2016). The CYP pathway also contributes to endogenous biosynthesis of the E‐series resolvins by producing the key intermediate 18‐HEPE (Arita et al., 2005). Indeed, we found 18‐HEPE in abundance within injured muscle of young mice but markedly deficient in aged mice. Therefore, metabolites of the LOX and CYP pathways are likely to act in unison. While we focused our intervention on a LOX‐derived resolvin, our data suggest that targeting the CYP pathway may be an additional novel strategy to combat basal age‐associated muscle inflammation and related dysfunction.

The ability of RvD1 to limit neutrophil infiltration is well‐described for certain experimental models of acute inflammation (Sun et al., 2007), but we have been unable to demonstrate this with sterile skeletal muscle injury (Markworth et al., 2020). The benefit of RvD1 on revascularization of ischemic skeletal muscle was recently found to be independent of effects on neutrophil infiltration (Sansbury et al., 2020). Therefore, the suppressive effects of RvD1 on neutrophil recruitment may depend on the nature of the inflammatory insult and the site of inflammation. A second defining action of specialized pro‐resolving mediators is accelerating a return to a non‐inflamed state by stimulating neutrophil clearance. Indeed, we previously found that RvD1 treatment reduced intramuscular neutrophil numbers by day 3 following muscle injury in young mice despite not limiting their initial appearance (Markworth et al., 2020). To our surprise, we found here that aged mice actually cleared these cells more rapidly than young mice and RvD1 treatment did not further accelerate the response. Neutrophils can inflict secondary muscle damage and limiting their influx is generally considered to be protective from muscle injury. Thus, the observation that aged muscle displayed both more rapid clearance of neutrophils and defective myofiber regeneration was surprising. Neutrophils do play an important role in muscular adaptations under certain conditions however and as such future studies are needed to better clarify the role of these cells in aging muscle (Lockhart & Brooks, 2008).

Consistent with recent reports, we found that aged mice displayed marked defects in regenerating myofiber number and size (Blanc et al., 2020; Patsalos et al., 2018; Rahman et al., 2020; Zhang et al., 2020), but RvD1 treatment had minimal impact on this response. Aging is also well‐established to limit recovery of muscle function following injury, due at least in part to an accumulation of fibrotic tissue during muscle regeneration (Rahman et al., 2020; Zhang et al., 2020). Indeed, we found that aged mice showed marked deficits in recovery of strength due to both reduced abilities of aged muscle to recover its pre‐injury size as well as maladaptive tissue remodeling that further impaired relative contractile function. Although treatment of aged mice with RvD1 did not impact any cellular indices of regenerating myofiber number/size or fiber type it did improve recovery of specific muscle force due to reduced accumulation of fibrotic tissue. Collectively, these data show that while RvD1 treatment appears unable to rescue age‐related defects in myofiber regeneration, it nonetheless limited maladaptive tissue remodeling and thus improved the quality of the regenerated muscle resulting in improved contractile function.

Our findings are in contrast to the beneficial effects of RvD1 treatment on regenerating muscle fiber size observed previously in young mice with this same dosing regimen (Markworth et al., 2020), and recent reports that local intramuscular injection of a distinct but related pro‐resolving mediator, resolvin D2 (RvD2), could also improve recovery of overall muscle size and strength (Giannakis et al., 2019). Additionally, RvD1 treatment stimulated revascularization of ischemic muscle in young mice (Sansbury et al., 2020). Consistent with our results are recent reports that systemic RvD1 treatment limited fibrosis of other tissues such as the heart (Hiram et al., 2020). Mice lacking the RvD1 receptor in all cell types (*Alx*/*Fpr2* ^−/−^ mice) or specifically in myeloid cells (hALX/FPR2^MKO^ mice) both display increased muscle fibrosis following hind‐limb ischemia (Sansbury et al., 2020). Thus, the age‐associated deficiency of endogenous ALX/FPR2 ligands (e.g., LXA_4_) during muscle regeneration and protective effect of exogenous RvD1 treatment (an ALX/FPR2 ligand) on maladaptive muscle remodeling identified in the current study are most likely mediated via intramuscular myeloid cells.

In conclusion, aging leads to a local deficiency of intramuscular pro‐resolving lipid mediator biosynthesis that is associated with chronic muscle inflammation, heightened acute myeloid cell responses to injury, and poor regenerative outcomes. Short‐term systemic treatment with RvD1 reduced local expression of inflammatory mediators and limited maladaptive tissue remodeling following muscle injury but was unable to fully overcome age‐associated defects in myofiber regeneration. Given their emerging important roles in stimulating tissue regeneration, other pro‐resolving mediators such as the E‐resolvins, protectins, and maresins, which were also deficient in aged muscle, may contribute to age‐related muscle dysfunction and warrant further investigation.

## 4 EXPERIMENTAL PROCEDURES

### 4.1 Animals

Aged female mice were obtained from the National Institute of Aging (NIA) at 20–22 months, housed for ~6 months, and used for experiments between 26–28 months. Adult **(**4–6 months) female C57BL/6 mice were obtained from Charles River Laboratories and served as young adult controls. All mice were housed under specific pathogen‐free conditions with ad‐libitum access to food and water.

### 4.2 Muscle injury

Mice were anesthetized with 2% isoflurane and received bilateral intramuscular injection of the tibialis anterior (TA) muscle with 50 µl per limb of 1.2% BaCl_2_ in sterile saline to induce myofiber injury. Mice were returned to their home cage to recover and monitored until ambulatory with free access to food and water.

### 4.3 Immunoresolvent treatment

RvD1 was purchased from Cayman Chemicals (10012554). Aged mice were randomized to receive daily 100 µl intraperitoneal (IP) injections of either 100 ng of RvD1 or vehicle control (0.1% ethanol) with the first dose given during ~5 min prior to muscle injury. Mice were allowed to recover for up to 2 weeks with daily IP injection of 100 ng of RvD1 or vehicle control.

### 4.4 MΦ phagocytosis

To confirm the established bioactivity of RvD1 on myeloid cells we performed in‐vitro phagocytosis assays using pHrodo Green *E*. *coli* Bio Particles (Invitrogen, P35366) with bone‐marrow‐derived macrophage (BMM) cultures isolated from young (4–6 months) and aged (26–28 months) host mice.

### 4.5 Histology

Cross‐sections (10 µm) were cut from the muscle mid‐belly in a cryostat at −20°C and adhered to SuperFrost Plus slides. Sections were air‐dried and then stained with hematoxylin and eosin (H & E). Slides for immune cell staining were fixed in acetone at −20°C and then air‐dried. Satellite cell staining slides were fixed in 4% paraformaldehyde (PFA), quenched with hydrogen peroxide, and antigen retrieval performed. Unfixed tissue sections were used for muscle fiber type staining. Prepared slides were blocked in 10% normal goat serum (Invitrogen 10000C) or Mouse on Mouse (M.O.M) blocking reagent (Vector Laboratories, MKB‐2213) as appropriate prior to overnight incubation at 4°C with primary antibodies. The following day, slides were incubated with Alexa Fluor conjugated secondary antibodies and mounted using Fluorescence Mounting Medium (Agilent Dako, S302380). Fluorescent images were captured using a Nikon A1 confocal microscope.

### 4.6 Image analysis

Muscle morphology was analyzed on stitched panoramic images of the entire muscle cross‐section by high‐throughput fully automated image analysis with the MuscleJ plugin for FIJI/ImageJ (Mayeuf‐Louchart et al., 2018). Immune cells and satellite cells were manually counted throughout the entire cross‐section and then normalized to tissue area as determined by MuscleJ. In all cases, the experimenter was blinded to the experimental group.

### 4.7 Muscle force testing

At day 14 post‐injury, maximum in‐s*itu* nerve‐stimulated isometric tetanic force (P_o_) generated by the TA muscle was measured and used to calculate maximal specific isometric force (sP_o_) by dividing P_o_ by muscle cross‐sectional area (CSA).

### 4.8 Mediator lipidomics

Muscle homogenates were analyzed by LC‐MS/MS based metabolipidomic profiling following solid‐phase extraction as previously described (Markworth et al., ,^2013^, ^2016^, ^2020^; Vella et al., 2019).

### 4.9 RT‐PCR

Whole muscle gene expression was measured by RT‐PCR on a CFX96 Real‐Time PCR Detection System (Bio‐Rad, 1855195) in 20 µL reactions of iTaq™ Universal SYBR^®^ Green Supermix (Bio‐Rad, #1725124) with 1 µM forward and reverse primers (Table 1). Relative mRNA expression was determined using the $2^{‐\Delta \Delta C_{t}}$ method. Primers are listed in Table 1.

**TABLE 1:** Real‐time PCR primers

Gene	Primer	Sequence
*Tnf1*	Forward	ATGGCCTCCCTCTCATCAGT
	Reverse	TGGTTTGCTACGACGTGGG
*Il6*	Forward	TCCGGAGAGGAGACTTCACA
	Reverse	TTGCCATTGCACAACTCTTTTCT
*Il1b*	Forward	GCCACCTTTTGACAGTGATGAG
	Reverse	GACAGCCCAGGTCAAAGGTT
*Ccl2*	Forward	AGCTGTAGTTTTTGTCACCAAGC
	Reverse	GACCTTAGGGCAGATGCAGT
*Il10*	Forward	GGCGCTGTCATCGATTTCTC
	Reverse	ATGGCCTTGTAGACACCTTGG
*Itgam*	Forward	TGGCCTATACAAGCTTGGCTTT
	Reverse	AAAGGCCGTTACTGAGGTGG
*Cd68*	Forward	ACTGGTGTAGCCTAGCTGGT
	Reverse	CCTTGGGCTATAAGCGGTCC
*Cd163*	Forward	TCTCCTGGTTGTAAAAGGTTTGT
	Reverse	CAGTTGTTTTCACCACCCGC
*Mrc1*	Forward	GGCTGATTACGAGCAGTGGA
	Reverse	CATCACTCCAGGTGAACCCC
*Adgre1*	Forward	CCAGGAGTGGAATGTCAAGATGT
	Reverse	GCAGACTGAGTTAGGACCACA
*Itgax*	Forward	GCAGACACTGAGTGATGCCA
	Reverse	TCGGAGGTCACCTAGTTGGG
*Actb*	Forward	CACTGTCGAGTCGCGTCC
	Reverse	TCATCCATGGCGAACTGGTG

### 4.10 Flow cytometry

Muscle tissue was digested with collagenase II/dispase. Isolated single‐cells were Fc blocked prior to incubation with fluorescently conjugated primary antibodies. Cells were run on a flow cytometer and data were analyzed with FlowJo 10 software.

### 4.11 Statistics

Data are presented as the mean ± SEM. Statistical analysis was performed in GraphPad Prism 7. Between‐group differences were tested by two‐tailed unpaired students t‐tests (2 groups) or by a one‐way analysis of variance (ANOVA) followed by pairwise Least Significance Difference (LSD) (3 groups) or Holm‐Sidak post hoc tests (>3 groups). *p *≤ 0.05 was used to determine statistical significance.

### 4.12 Study approval

All animal experiments were approved by the University of Michigan Institutional Animal Care and Use committee (IACUC) (PRO00008744).

## CONFLICT OF INTEREST

The authors declare that there are no conflicts of interest.

## AUTHOR CONTRIBUTIONS

J.F.M. and S.V.B. conceived the study. S.V.B., K.R.M., P.C.D.M., and C.A.A. supervised the work. J.F.M., L.A.B., C.A.A., and S.V.B. designed the experiments. J.F.M., L.A.B., E.L., J.L., J.A.C.‐M., and C.D. performed the experiments. J.F.M., L.A.B., J.L., and K.R.M. analyzed the data. J.F.M. prepared the figures and wrote the manuscript with input from all authors.

## Supporting information

Fig S1‐S4

Table S1

Table S2

Supplementary Material

## Data Availability

Original data will be made available upon request.
