# Supplementary material for: Metabolipidomic profiling reveals an age‐related deficiency of skeletal muscle pro‐resolving mediators that contributes to maladaptive tissue remodeling
Source: Aging Cell. 2021 Jun 2;20(6):e13393. doi: 10.1111/acel.13393 (PMC8208786; doi:10.1111/acel.13393)
Supplement: Supplementary file 1 — Fig S1‐S4 [file ACEL-20-e13393-s004.pdf]

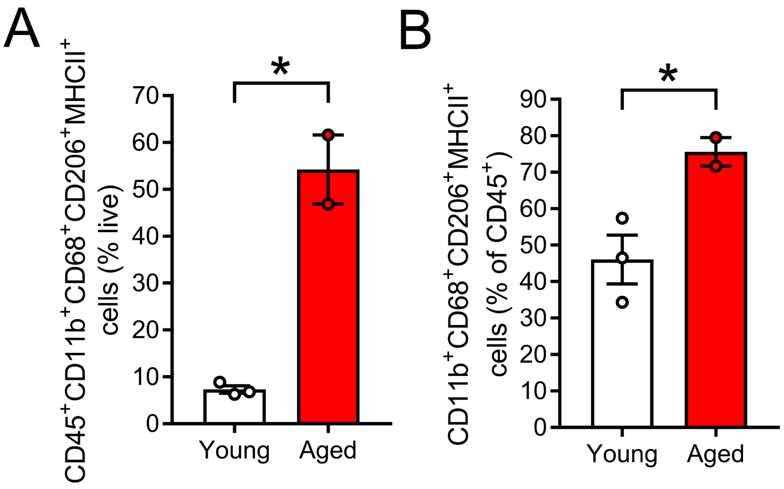

Supplemental Figure 1 – Flow cytometry analysis of basal myeloid cell accumulation in aged skeletal muscle: Single-cells isolated from pooled hind-limb muscles obtained from young and aged female C57BL/6 mice were analyzed by flow cytometry using antibodies including CD45-BV785, CD11b-PE, CD68-FITC, CD206-PE/Dazzle 594, MHCII (I-A/I-E)-APC/Cyanine 7. Dead cells were excluded from analysis with Propidium iodide (PI). **A:** M2-like resident muscle macrophages (defined as $CD45^{+}CD11b^{+}CD68^{+}CD206^{+}MHCII^{+}$ cells) expressed as percentage of the total live cell population. **B:** M2-like resident muscle macrophages ($CD11b^{+}CD68^{+}CD206^{+}MHCII^{+}$ cells) expressed as percentage of total intramuscular leukocytes ($CD45^{+}$ cells). Bars show mean ± SEM of 2-3 mice per group with dots representing data from each individual mouse. *Denotes $p<0.05$ vs vehicle group by a two-tailed unpaired t-test.

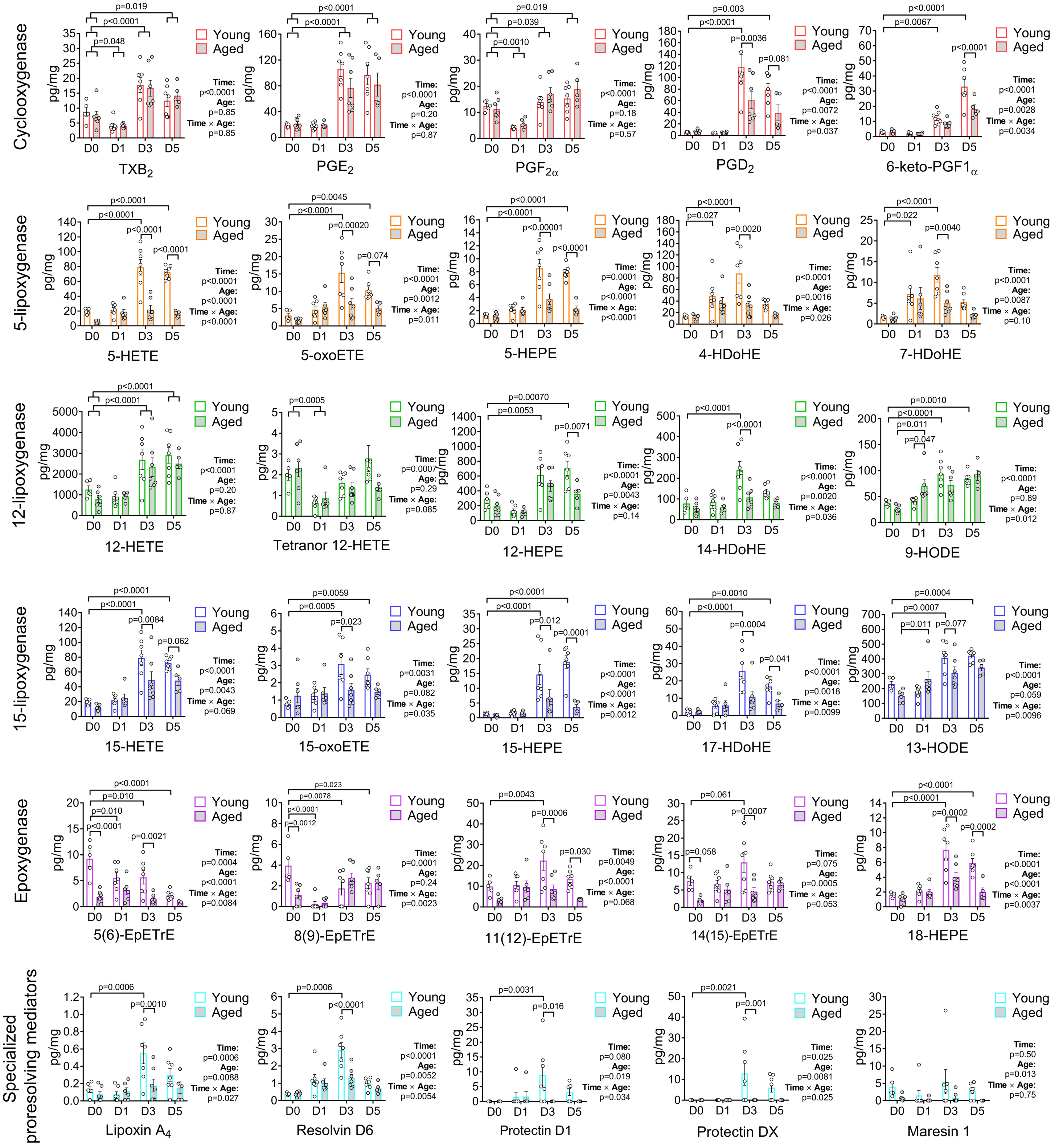

Supplemental Figure 2 – Impact of age on local shifts in lipid mediator biosynthesis following muscle injury: Young and aged female C57BL/6 mice received bilateral intramuscular injection of the TA muscle with 50 µL of 1.2% $BaCl_2$ to induce myofiber injury. TA muscles were collected at day 1 (D1), day 3 (D3), and day 5 (D5) post-injury for analysis of intramuscular lipid mediator concentrations via LC-MS/MS. Age-matched uninjured TA muscles served as control samples (D0). Bars show the mean ± SEM of 5-7 mice per group with dots representing data from each individual mouse. P-values were determined by two-way ANOVA with Holm-Sidak post-hoc tests vs. age-matched uninjured mice and for aged vs. young mice at each time-point.

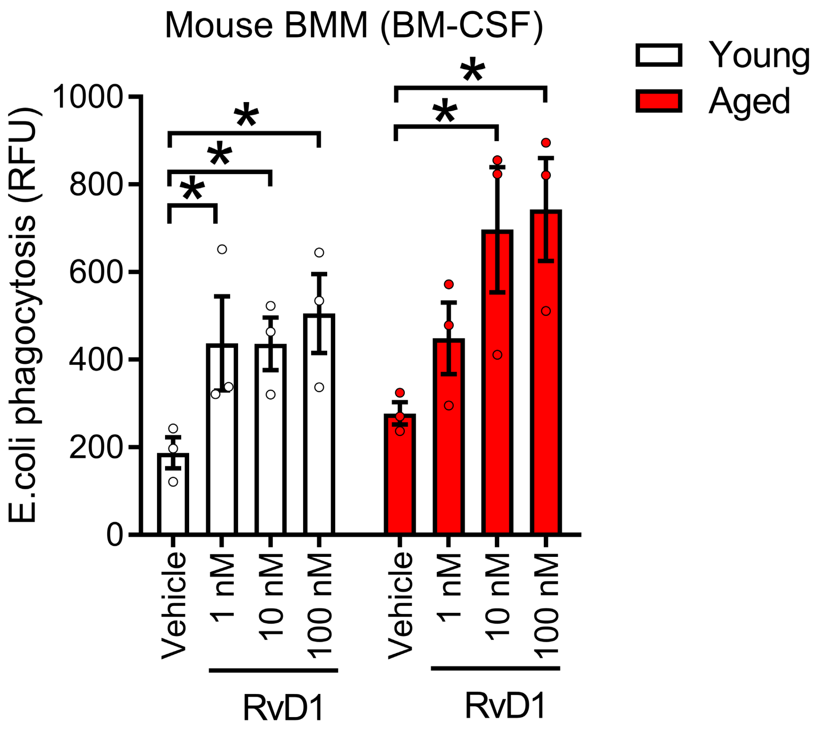

Supplemental Figure 3 – Resolvin D1 treatment stimulates phagocytosis in macrophages from both young and aged hosts: Bone marrow-derived macrophages (BMMs) from young and aged C57BL/6 mice were obtained by culturing myeloid precursor cells for 7 days in the presence of 20 ng/mL GM-CSF. GM-CSF derived BMMs were then pre-treated for 15 minutes with resolvin D1 (RvD1, 1-100 nM) prior to incubation with pHrodo Green *E. Coli* Bio Particles for 60 min at 37°C. Phagocytosis was quantified as the fluorescence intensity in relative fluorescence units (RFU) as determined by measurement of the entire well with a plate reader. Bars show the mean ± SEM of cells from three young and three aged donor mice for each experimental condition. *Denotes $p<0.05$ by two-way ANOVA with Holm-Sidak post-hoc tests vs. aged-matched vehicle treated BMMs.

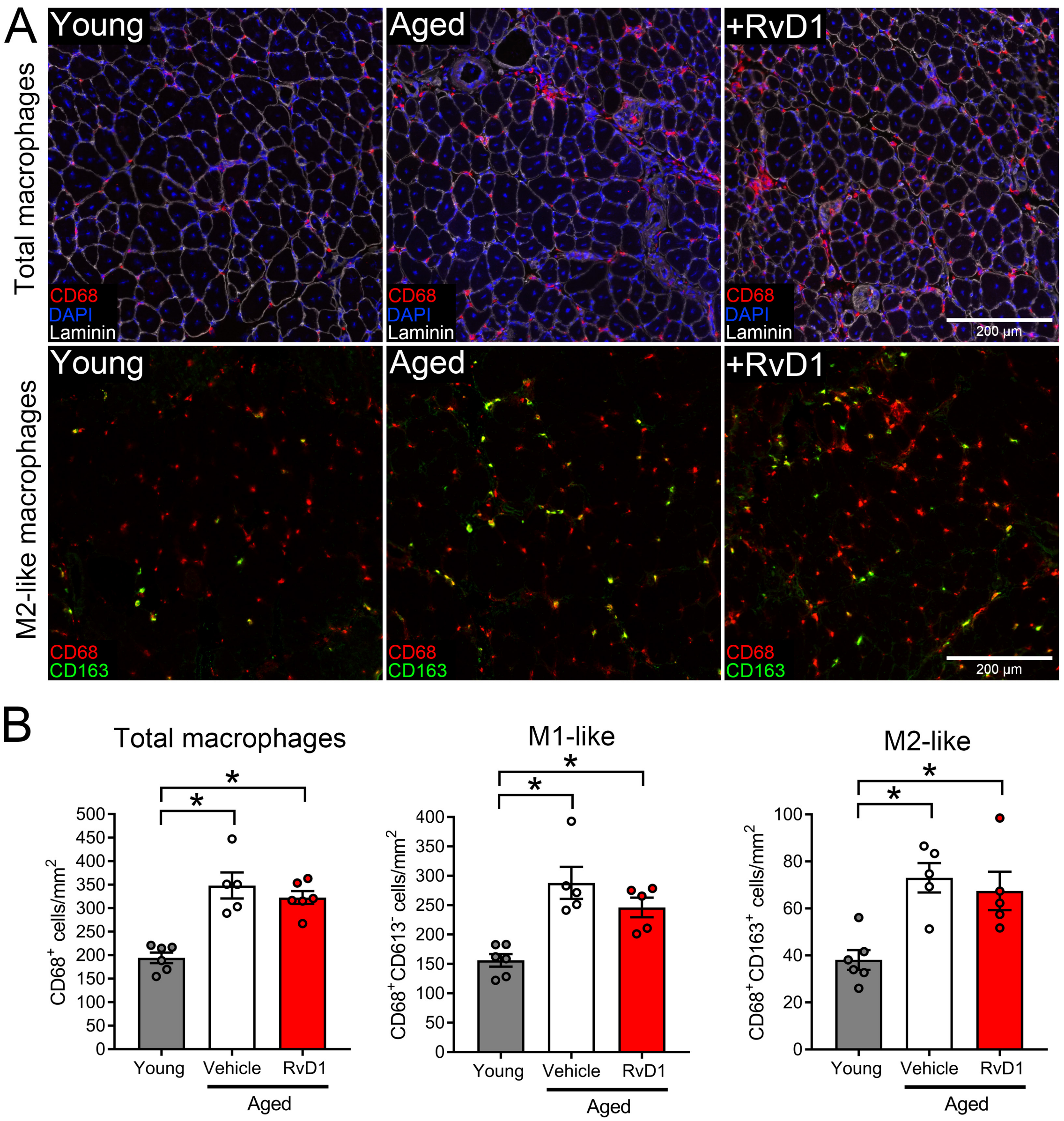

Supplemental Figure 4 – Resolvin D1 does not impact upon persistent elevation of intramuscular macrophages following muscle injury in aged mice. A: Young and aged C57BL/6 mice received bilateral intramuscular injection of the TA muscle with 50 µL of 1.2% $BaCl_2$ to induce myofiber injury. Aged mice were then treated with daily intraperitoneal injections of resolvin D1 (RvD1, 100 ng) or vehicle (0.1% ethanol) for 14 days. TA muscles were collected at day 14 post-injury and tissue cross-sections were stained for total macrophages (MΦ, CD68), or M2-like MΦ (CD163). Cell nuclei and the basal lamina were counterstained with DAPI and a laminin antibody respectively. Scale bars are 200 µm. **B:** Quantification of myeloid cells in regenerating muscle including total MΦ (CD68$^+$ cells), M1-like MΦ (CD68$^+$CD163$^-$ cells), and M2-like MΦ (CD68$^+$CD163$^+$ cells). Bars show mean ± SEM of 5-6 mice per group with dots representing data from each individual mouse. Denotes *p<0.05 by one-way ANOVA with pairwise LSD post-hoc tests.
