# Supplementary material for: Metabolipidomic profiling reveals an age‐related deficiency of skeletal muscle pro‐resolving mediators that contributes to maladaptive tissue remodeling
Source: Aging Cell. 2021 Jun 2;20(6):e13393. doi: 10.1111/acel.13393 (PMC8208786; doi:10.1111/acel.13393)
Supplement: Supplementary file 2 — Table S1 [file ACEL-20-e13393-s002.pdf]

Supplemental Table 1A: Cyclooxygenase (COX) metabolite concentration (pmol/g) in young and aged mouse tibialis anterior (TA) muscle homogenates

Pathway	Substrate	Analyte	Uninjured		Day 1 post-injury		Day 3 post-injury		Day 5 post-injury	
			Young	Aged	Young	Aged	Young	Aged	Young	Aged
Cyclooxygenase	20:3n-6	PGE1	ND	ND	0.70 ± 0.27	ND	13.88 ± 1.95	3.32 ± 1.42	8.43 ± 1.06	2.28 ± 1.10
	20:3n-6	PGF1a	1.31 ± 0.13	0.98 ± 0.05	ND	0.57 ± 0.20	1.81 ± 0.37	1.55 ± 0.11	1.48 ± 0.40	1.86 ± 0.23
	20:3n-6	15-keto PGE1	ND	ND	ND	ND	ND	ND	ND	ND
	20:3n-6	13,14dhPGE1	ND	ND	ND	ND	ND	ND	ND	ND
	20:3n-6	13,14dh-15k-PGE1	ND	ND	ND	ND	0.64 ± 0.18	0.82 ± 0.18	0.50 ± 0.23	0.81 ± 0.21
	20:3n-6	D17-PGE1	ND	ND	ND	ND	ND	ND	ND	ND
	20:3n-6	Bicyclo PGE1	ND	ND	ND	ND	0.36 ± 0.17	0.69 ± 0.19	ND	ND
	20:3n-6	6-keto PGE1	ND	ND	ND	ND	ND	ND	ND	ND
	20:3n-6	2,3-dinor PGE1	ND	ND	ND	ND	ND	ND	ND	ND
	20:3n-6	19(R)-hydroxy PGE1	ND	ND	ND	ND	ND	ND	ND	ND
	20:3n-6	15(R)-PGE1	ND	ND	ND	ND	ND	ND	ND	ND
	20:4n-6	TXB2	23.79 ± 4.11	19.89 ± 4.22	11.22 ± 2.11	11.00 ± 0.83	47.99 ± 6.79	45.00 ± 7.23	33.80 ± 5.24	38.23 ± 4.42
	20:4n-6	12(S)-HHTrE	49.09 ± 3.07	40.30 ± 5.71	29.65 ± 3.30	41.25 ± 3.49	134.23 ± 17.98	149.24 ± 28.94	152.13 ± 12.56	170.12 ± 30.79
	20:4n-6	PGD2	15.33 ± 0.73	17.82 ± 3.24	11.36 ± 1.01	12.74 ± 1.58	334.51 ± 63.33	172.63 ± 41.49	224.40 ± 30.18	110.94 ± 39.28
	20:4n-6	PGE2	53.74 ± 4.05	62.08 ± 9.08	51.75 ± 5.44	54.04 ± 4.74	300.41 ± 32.21	218.60 ± 42.19	274.60 ± 42.63	233.04 ± 50.86
	20:4n-6	PGF2a	35.31 ± 2.51	31.88 ± 4.28	11.30 ± 0.53	15.77 ± 2.14	39.65 ± 5.84	48.36 ± 6.56	43.27 ± 5.68	53.38 ± 8.42
	20:4n-6	6kPGF1a	7.34 ± 0.86	7.61 ± 1.23	5.04 ± 0.74	4.91 ± 0.43	32.99 ± 4.58	22.00 ± 2.61	89.12 ± 12.16	49.26 ± 7.31
	20:4n-6	PGJ2	0.70 ± 0.19	0.92 ± 0.20	1.14 ± 0.13	0.99 ± 0.22	28.25 ± 3.31	15.70 ± 3.73	30.49 ± 6.41	15.11 ± 5.19
	20:4n-6	PGA2	0.24 ± 0.21	0.99 ± 0.50	1.24 ± 0.41	1.51 ± 0.35	18.22 ± 7.40	12.79 ± 5.96	23.62 ± 9.07	13.57 ± 6.58
	20:4n-6	15-keto PGE2	3.77 ± 0.52	3.56 ± 0.66	3.70 ± 0.55	3.34 ± 0.60	12.51 ± 1.88	6.40 ± 1.48	10.71 ± 1.94	6.94 ± 1.95
	20:4n-6	15-keto PGF2a	ND	ND	ND	ND	9.02 ± 1.61	4.61 ± 1.20	6.17 ± 0.92	3.39 ± 1.14
	20:4n-6	13,14dh-15k-PGE2	4.02 ± 0.37	4.49 ± 0.26	ND	ND	13.12 ± 1.58	9.00 ± 1.62	6.65 ± 2.56	10.22 ± 2.01
	20:4n-6	13,14dh-15k-PGD2	4.56 ± 0.70	4.39 ± 1.04	2.84 ± 0.58	1.87 ± 0.14	21.03 ± 3.42	7.56 ± 1.68	19.02 ± 3.27	7.22 ± 2.12
	20:4n-6	19(R)-OH PGF2a & 20-OH PGF2a	0.61 ± 0.08	0.61 ± 0.05	0.79 ± 0.12	0.46 ± 0.45	0.71 ± 0.12	0.46 ± 0.13	0.96 ± 0.23	0.59 ± 0.15
	20:4n-6	8-isoPGF2a & 11bPGF2a	1.10 ± 0.05	1.14 ± 0.25	0.30 ± 0.13	0.94 ± 0.07	1.89 ± 0.53	3.22 ± 0.51	2.54 ± 0.38	3.11 ± 0.75
	20:4n-6	11dh-2,3-dinor TXB2	2.42 ± 0.38	1.36 ± 0.48	ND	ND	ND	ND	ND	ND
	20:4n-6	D12-PGJ2	1.07 ± 0.11	1.15 ± 1.15	0.62 ± 0.16	0.63 ± 0.28	12.87 ± 5.92	8.03 ± 2.57	9.76 ± 3.14	3.76 ± 2.46
	20:4n-6	tetranor PGEM	ND	ND	ND	ND	ND	ND	ND	ND
	20:4n-6	15d-D12,14-PGJ2	ND	ND	ND	ND	ND	ND	ND	ND
	20:4n-6	Bicyclo PGE2	ND	ND	ND	ND	1.49 ± 0.14	0.66 ± 0.25	ND	ND
	20:4n-6	19(R)-OH PGE2 & 20-OH PGE2	ND	ND	ND	ND	ND	ND	ND	ND
	20:4n-6	2,3-dinor TXB2	ND	ND	ND	ND	ND	ND	ND	ND
	20:4n-6	11dh-TXB2	ND	ND	ND	ND	ND	ND	ND	ND
	20:4n-6	13,14dh-15k-PGF2a	ND	ND	ND	ND	ND	ND	ND	ND
	20:4n-6	6,15-diketo PGFa	ND	ND	ND	ND	ND	ND	ND	ND
	20:4n-6	iPF-VI	ND	ND	ND	ND	ND	ND	ND	ND
	20:5n-3	TXB3	2.93 ± 0.71	1.86 ± 0.42	0.51 ± 0.22	ND	4.21 ± 1.20	2.57 ± 0.24	3.75 ± 0.87	1.25 ± 0.39
	20:5n-3	PGE3	0.67 ± 0.18	0.82 ± 0.25	0.39 ± 0.19	ND	6.80 ± 0.96	3.62 ± 0.68	5.43 ± 1.07	2.76 ± 0.94
	20:5n-3	PGF3a	0.48 ± 0.03	0.56 ± 0.04	ND	ND	ND	0.72 ± 0.07	ND	ND
	20:5n-3	PGD3	ND	ND	ND	ND	4.98 ± 1.41	ND	3.58 ± 0.88	ND
	20:5n-3	11dh TXB3	ND	ND	ND	ND	ND	ND	ND	ND
	20:5n-3	15d-D12,14-PGJ3	ND	ND	ND	ND	ND	ND	ND	ND
		Sum	209.57 ± 11.59	204.25 ± 29.84	136.63 ± 13.24	153.00 ± 12.19	1043.67 ± 145.99	740.86 ± 139.65	954.35 ± 125.70	730.84 ± 157.98
		Δ change	0.00 11.59	0.00 29.84	-72.95 ± 13.24	-51.25 ± 12.19	834.10 ± 145.99	536.61 ± 139.65	744.77 ± 125.70	526.59 ± 157.98

Values are mean ± SEM of 5-7 mice/group. ND = Below limits of detection of the assay in ≥ 50% of samples. Remaining missing values were replaced with half the minimum positive value in the original data set.

Relative concentration

Low High

Supplemental Table 1B: Lipoxygenase (LOX) metabolite concentration (pmol/g) in young and aged mouse tibialis anterior (TA) muscle homogenates

			Uninjured		Day 1 post-injury		Day 3 post-injury		Day 5 post-injury	
Pathway	Substrate	Analyte	Young	Aged	Young	Aged	Young	Aged	Young	Aged
5-LOX	18:3n-3	9(S)-HOTrE	16.09 ± 1.42	6.35 ± 0.77	6.28 ± 1.20	8.37 ± 2.34	10.97 ± 1.56	8.12 ± 1.70	15.49 ± 1.34	6.39 ± 0.70
	18:3n-3	9-OxoOTrE	8.56 ± 0.65	3.73 ± 0.65	2.78 ± 0.38	4.62 ± 1.65	6.25 ± 1.22	3.93 ± 0.77	10.00 ± 1.34	4.80 ± 0.70
	20:3n-9	5(S)-HETrE	0.71 ± 0.04	0.34 ± 0.03	0.65 ± 0.11	0.70 ± 0.23	2.48 ± 0.41	1.25 ± 0.34	1.74 ± 0.14	0.83 ± 0.08
	20:4n-6	5-HETE	28.97 ± 4.50	15.67 ± 2.13	52.83 ± 9.47	55.51 ± 13.26	150.88 ± 24.12	67.85 ± 17.31	115.65 ± 4.79	49.63 ± 5.57
	20:4n-6	5-oxoETE	9.51 ± 2.07	5.03 ± 0.71	14.84 ± 3.19	16.94 ± 4.13	48.29 ± 8.96	19.91 ± 5.30	32.23 ± 3.25	15.99 ± 2.24
	20:4n-6	LTB4	ND	ND	ND	ND	ND	ND	ND	ND
	20:4n-6	12-OxoLTB4	0.86 ± 0.29	ND	0.67 ± 0.20	0.70 ± 0.10	2.35 ± 0.76	1.44 ± 0.69	1.86 ± 0.76	ND
	20:5n-3	5-HEPE	3.87 ± 0.32	3.19 ± 0.62	7.06 ± 1.08	6.68 ± 1.21	26.82 ± 4.32	11.83 ± 2.59	24.95 ± 1.28	7.19 ± 1.42
	20:5n-3	LTB5	1.35 ± 0.44	1.16 ± 0.30	ND	ND	1.20 ± 0.55	ND	ND	ND
	22:6n-3	4-HDoHE	38.45 ± 3.36	35.30 ± 4.54	141.37 ± 35.53	105.76 ± 28.34	255.40 ± 48.20	92.50 ± 22.30	102.08 ± 8.47	43.73 ± 4.09
	22:6n-3	7-HDoHE	4.57 ± 0.40	2.63 ± 0.57	20.99 ± 5.65	17.91 ± 7.63	34.47 ± 5.13	14.55 ± 2.84	15.21 ± 2.18	6.27 ± 1.18
	5-LOX sum		112.97 ± 9.68	73.67 ± 9.24	247.76 ± 53.34	217.39 ± 58.35	539.12 ± 91.11	221.65 ± 51.52	319.95 ± 17.75	135.76 ± 13.61
	5-LOX Δ change		0.00 ± 9.68	0.00 ± 9.24	134.79 ± 53.34	143.72 ± 58.35	426.15 ± 91.11	147.98 ± 51.52	206.98 ± 17.75	62.09 ± 13.61
8-LOX	20:4n-6	8-HETE	19.22 ± 1.16	7.59 ± 2.00	25.25 ± 3.87	29.35 ± 10.90	71.27 ± 12.40	40.53 ± 9.82	66.69 ± 5.88	32.88 ± 4.48
	20:5n-3	8-HEPE	3.70 ± 0.27	2.03 ± 0.56	4.71 ± 0.74	3.73 ± 0.82	14.38 ± 2.14	8.01 ± 1.33	13.31 ± 1.07	4.44 ± 0.90
	8-LOX sum		22.92 ± 1.35	9.61 ± 2.39	29.96 ± 4.50	33.08 ± 11.70	85.65 ± 14.46	48.53 ± 10.97	80.01 ± 6.72	37.33 ± 5.26
	8-LOX Δ change		0.00 ± 1.35	0.00 ± 2.39	7.04 ± 4.50	23.46 ± 11.70	62.73 ± 14.46	38.92 ± 10.97	57.09 ± 6.72	27.71 ± 5.26
12-LOX	18:2n-6	9-HODE	125.00 ± 8.72	87.54 ± 8.42	140.06 ± 9.47	238.76 ± 44.01	318.56 ± 36.40	243.55 ± 31.30	283.17 ± 15.76	306.88 ± 33.91
	18:2n-6	9-OxoODE	116.73 ± 14.06	100.13 ± 15.23	122.49 ± 24.19	170.24 ± 51.30	245.22 ± 34.18	144.06 ± 23.25	217.42 ± 34.53	192.93 ± 28.54
	20:3n-6	8(S)-HETrE	5.83 ± 0.70	2.57 ± 0.50	5.50 ± 0.97	4.64 ± 0.82	21.33 ± 3.34	7.06 ± 1.19	14.66 ± 1.46	6.00 ± 0.79
	20:2n-6	11(R)-HEDE	3.12 ± 0.15	2.53 ± 0.34	3.46 ± 0.49	4.39 ± 0.64	25.96 ± 2.56	13.04 ± 2.77	17.68 ± 1.29	8.95 ± 1.08
	20:4n-6	12-HETE	3924.38 ± 557.26	2479.13 ± 492.73	2774.45 ± 564.56	2855.33 ± 250.09	8393.85 ± 1483.68	7283.21 ± 1413.43	9073.93 ± 1200.48	7809.81 ± 761.53
	20:4n-6	5(S),12(S)-DiHETE	78.12 ± 17.20	41.62 ± 9.71	35.59 ± 8.71	30.62 ± 6.28	103.23 ± 18.26	86.97 ± 11.37	89.72 ± 16.71	82.18 ± 20.92
	20:4n-6	tetranor 12-HETE	7.53 ± 1.00	8.68 ± 1.53	2.39 ± 0.44	3.26 ± 1.13	6.02 ± 0.76	5.22 ± 0.96	10.45 ± 2.34	5.22 ± 0.70
	20:4n-6	12-OxoETE	ND	ND	ND	ND	ND	ND	ND	ND
	20:5n-3	12-HEPE	908.14 ± 184.26	631.22 ± 153.89	406.04 ± 86.99	353.26 ± 58.46	1939.05 ± 316.59	1462.73 ± 225.65	2212.58 ± 303.91	1134.75 ± 197.87
	22:6n-3	14-HDoHE	226.44 ± 56.59	149.60 ± 33.80	246.42 ± 46.97	170.44 ± 29.11	698.24 ± 116.18	312.73 ± 55.20	393.04 ± 34.31	232.19 ± 26.10
	12-LOX sum		5406.54 ± 804.62	3503.05 ± 665.86	3736.42 ± 701.44	3830.95 ± 342.85	11751.48 ± 1951.27	9558.61 ± 1722.33	12312.69 ± 1507.16	9778.96 ± 894.38
	12-LOX sum (No HODEs)		5164.82 ± 809.65	3315.38 ± 664.45	3473.87 ± 694.29	3421.95 ± 313.29	11187.71 ± 1909.09	9171.00 ± 1681.06	11812.11 ± 1537.53	9279.14 ± 890.25
	12-LOX Δ change		0.00 ± 804.62	0.00 ± 665.86	-1670.13 ± 701.44	327.90 ± 342.85	6344.94 ± 1951.27	6055.56 ± 1722.33	6906.15 ± 1507.16	6275.90 ± 894.38
	12-LOX Δ change (No HODEs)		0.00 ± 809.65	0.00 ± 664.45	-1690.95 ± 694.29	106.57 ± 313.29	6022.89 ± 1909.09	5855.61 ± 1681.06	6647.29 ± 1537.53	5963.75 ± 890.25
15-LOX	18:2n-6	13-HODE	783.59 ± 59.04	507.78 ± 47.51	589.22 ± 52.72	897.92 ± 175.07	1378.36 ± 138.36	1037.86 ± 132.62	1424.11 ± 51.96	1153.73 ± 76.43
	18:2n-6	13-OxoODE	226.73 ± 15.31	148.99 ± 15.04	224.25 ± 30.18	383.41 ± 110.22	521.44 ± 74.08	306.15 ± 40.31	392.42 ± 47.48	302.48 ± 16.50
	18:3n-3	13(S)-HOTrE	17.51 3.38	5.80 1.38	6.79 0.85	8.27 1.81	11.31 # 1.78	9.22 1.33	24.63 1.71	14.45 1.97
	18:3n-6	13(S)-HOTrE(g)	1.60 ± 0.97	0.80 ± 0.50	ND	ND	2.90 ± 1.13	4.18 ± 1.69	5.82 ± 2.14	7.63 ± 2.22
	20:2n-6	15(S)-HEDE	1.07 ± 0.10	0.66 ± 0.07	0.94 ± 0.17	1.08 ± 0.28	6.16 ± 0.80	2.73 ± 0.56	4.17 ± 0.23	2.10 ± 0.18
	20:2n-6	15-OxoEDE	ND	ND	0.51 ± 0.23	0.76 ± 0.20	3.07 ± 0.65	1.45 ± 0.27	1.37 ± 0.36	ND
	20:4n-6	15-HETE	60.96 ± 5.16	37.17 ± 4.12	67.82 ± 9.29	77.52 ± 16.72	246.84 ± 32.90	152.81 ± 35.38	226.17 ± 10.10	150.88 ± 20.75
	20:4n-6	15-OxoETE	2.48 ± 0.33	3.95 ± 1.31	3.92 ± 0.67	4.37 ± 1.08	8.31 ± 2.12	5.04 ± 1.15	7.77 ± 1.06	4.65 ± 0.47
	20:4n-6	8(S),15(S)-DiHETE	0.52 ± 0.15	ND	ND	ND	ND	ND	0.72 ± 0.27	ND
	20:4n-6	5(S),15(S)-DiHETE	ND	ND	ND	ND	ND	ND	ND	ND
	20:5n-3	15(S)-HEPE	4.35 ± 0.62	2.14 ± 0.56	5.40 ± 0.86	4.19 ± 0.79	45.85 ± 10.55	21.18 ± 8.74	59.15 ± 6.59	11.34 ± 2.69
	20:5n-3	5(S),15(S)-DiHEPE	0.96 ± 0.30	0.36 ± 0.10	ND	ND	ND	ND	ND	ND
	22:6n-3	17-HDoHE	6.49 ± 1.79	6.11 ± 0.90	16.30 ± 3.97	16.99 ± 7.57	74.49 ± 12.93	30.78 ± 10.05	48.73 ± 5.73	20.06 ± 5.11
	15-LOX sum		1106.38 ± 62.22	713.96 ± 65.21	916.66 ± 93.14	1398.70 ± 310.55	2298.85 ± 254.20	1572.02 ± 215.04	2195.54 ± 99.51	1668.32 ± 115.12
	15-LOX sum (No HODEs)		96.07 ± 3.80	57.18 ± 5.58	103.18 ± 12.88	117.37 ± 28.17	399.04 ± 50.31	228.01 ± 55.43	379.01 ± 17.73	212.10 ± 28.21
	15-LOX Δ change		0.00 ± 62.22	0.00 ± 65.21	-189.73 ± 93.14	684.74 ± 310.55	1192.46 ± 254.20	858.06 ± 215.04	1089.15 ± 99.51	954.36 ± 115.12
	15-LOX Δ change (No HODEs)		0.00 ± 3.80	0.00 ± 5.58	7.11 ± 12.88	60.19 ± 28.17	302.97 ± 50.31	170.83 ± 55.43	282.94 ± 17.73	154.92 ± 28.21
Total LOX sum			6648.82 ± 854.63	4300.29 ± 713.78	4930.80 ± 791.11	5480.11 ± 618.52	14675.10 ± 2206.36	11400.81 ± 1968.10	14908.18 ± 1474.97	11620.36 ± 1002.30
Total LOX Δ change			0.00 ± 854.63	0.00 ± 713.78	-1718.02 ± 791.11	1179.82 ± 618.52	8026.28 ± 2206.36	7100.51 ± 1968.10	8259.37 ± 1474.97	7320.06 ± 1002.30

Values are mean ± SEM of 5-7 mice/group. ND = Below limits of detection of the assay in ≥ 50% of samples. Remaining missing values were replaced with half the minimum positive value in the original data set.

Relative concentration

Low High

Supplemental Table 1C: Epoxygenase (CYP) metabolite concentration (pmol/g) in young and aged mouse tibialis anterior (TA) muscle homogenates

Pathway	Substrate	Analyte	Uninjured		Day 1 post-injury		Day 3 post-injury		Day 5 post-injury	
			Young	Aged	Young	Aged	Young	Aged	Young	Aged
Epoxygenase	18:2n-6	9(10)-EpOME	408.59 ± 23.09	64.95 ± 31.97	239.46 ± 35.10	182.58 ± 119.88	220.98 ± 78.28	140.80 ± 30.86	253.68 ± 51.27	97.70 ± 24.91
	18:2n-6	12(13)-EpOME	163.87 ± 13.84	68.58 ± 15.18	119.19 ± 16.32	149.20 ± 45.28	166.91 ± 24.29	84.81 ± 11.25	131.71 ± 13.96	60.67 ± 3.46
	18:2n-6	9,10-DiHOME	19.13 ± 1.71	15.45 ± 3.64	20.02 ± 1.64	20.13 ± 1.82	24.89 ± 2.42	25.38 ± 2.72	35.37 ± 2.52	27.37 ± 2.47
	18:2n-6	12,13-DiHOME	16.14 ± 1.18	15.33 ± 3.25	21.01 ± 2.19	20.21 ± 1.68	22.55 ± 2.36	25.21 ± 2.87	41.80 ± 4.61	31.68 ± 3.01
	20:4n-6	5(6)-EpETrE	28.92 ± 4.65	5.22 ± 1.57	17.36 ± 3.20	9.93 ± 2.72	17.66 ± 4.00	4.58 ± 1.09	6.82 ± 1.02	2.54 ± 0.24
	20:4n-6	8(9)-EpETrE	12.36 ± 2.50	3.47 ± 1.07	ND	ND	5.46 ± 1.97	8.65 ± 1.41	7.05 ± 1.64	7.14 ± 1.96
	20:4n-6	11(12)-EpETrE	30.90 ± 5.63	8.78 ± 2.37	32.86 ± 5.79	30.22 ± 8.95	69.86 ± 14.58	26.71 ± 6.47	41.95 ± 5.06	11.15 ± 0.47
	20:4n-6	14(15)-EpETrE	23.76 ± 3.92	5.33 ± 1.28	20.85 ± 3.64	16.06 ± 4.91	40.56 ± 8.87	14.24 ± 3.54	23.20 ± 3.32	20.29 ± 2.03
	20:4n-6	5,6-DiHETrE	0.10 ± 0.05	0.12 ± 0.04	0.41 ± 0.12	0.51 ± 0.20	0.22 ± 0.06	0.26 ± 0.09	0.40 ± 0.11	0.48 ± 0.12
	20:4n-6	8,9-DiHETrE	0.64 ± 0.06	0.38 ± 0.08	0.57 ± 0.07	0.59 ± 0.12	0.85 ± 0.16	0.54 ± 0.11	0.69 ± 0.14	0.43 ± 0.18
	20:4n-6	11,12-DiHETrE	3.24 ± 0.28	2.17 ± 0.42	2.38 ± 0.28	0.59 ± 0.15	4.32 ± 0.53	3.63 ± 0.34	4.04 ± 0.43	3.57 ± 0.49
	20:4n-6	14,15-DiHETrE	4.24 ± 0.34	3.39 ± 0.69	3.92 ± 0.40	3.69 ± 0.32	6.32 ± 0.83	5.56 ± 0.30	6.45 ± 0.58	6.13 ± 0.53
	20:4n-6	20-HETE	33.22 ± 4.85	10.62 ± 3.14	67.70 ± 13.00	64.39 ± 25.45	128.63 ± 29.79	40.66 ± 12.16	66.12 ± 11.99	3.93 ± 1.26
	20:5n-3	8(9)-EpETE	1.06 ± 0.13	ND	1.07 ± 0.20	0.79 ± 0.27	3.56 ± 0.98	1.08 ± 0.45	1.81 ± 0.52	ND
	20:5n-3	11(12)-EpETE	1.33 ± 0.38	ND	1.06 ± 0.41	0.66 ± 0.33	3.50 ± 0.78	ND	1.79 ± 0.91	ND
	20:5n-3	14(15)-EpETE	2.94 ± 1.29	ND	2.45 ± 0.50	3.05 ± 1.17	7.52 ± 1.41	3.79 ± 1.19	4.87 ± 1.46	2.81 ± 0.76
	20:5n-3	17(18)-EpETE	2.96 ± 0.41	0.93 ± 0.38	2.71 ± 0.34	2.05 ± 0.49	7.86 ± 1.30	3.27 ± 1.03	6.65 ± 0.68	1.45 ± 0.48
	20:5n-3	18-HEPE	5.07 ± 0.46	3.04 ± 0.75	6.79 ± 0.95	6.18 ± 1.14	24.15 ± 3.44	12.59 ± 1.96	18.57 ± 1.93	6.28 ± 1.78
	22:6n-3	7(8)-EpDPE	8.69 ± 0.51	4.18 ± 1.21	19.33 ± 5.23	12.47 ± 4.45	27.94 ± 6.40	8.34 ± 2.08	9.14 ± 1.24	2.13 ± 0.25
	22:6n-3	10(11)-EpDPE	32.30 ± 2.20	14.76 ± 4.37	78.58 ± 21.26	49.95 ± 18.85	113.51 ± 27.93	30.05 ± 6.58	36.16 ± 5.42	7.48 ± 0.68
	22:6n-3	13(14)-EpDPE	16.57 ± 1.31	8.09 ± 2.22	37.85 ± 9.65	23.05 ± 7.43	46.06 ± 11.33	16.44 ± 3.13	19.30 ± 2.69	5.49 ± 0.64
	22:6n-3	16(17)-EpDPE	12.17 ± 0.97	5.99 ± 1.55	26.87 ± 6.62	16.46 ± 5.37	41.27 ± 8.21	12.49 ± 2.10	14.61 ± 1.56	3.94 ± 0.37
	22:6n-3	19(20)-EpDPE	15.74 ± 0.83	8.44 ± 2.14	46.68 ± 13.82	24.61 ± 8.13	63.45 ± 9.84	35.18 ± 6.33	31.26 ± 1.56	13.82 ± 0.50
		Sum	843.95 ± 50.37	250.88 ± 61.51	769.90 ± 122.68	396.97 ± 21.18	1048.07 ± 168.20	504.70 ± 61.55	763.45 ± 88.98	319.06 ± 27.03
		Sum (no EpOMEs)	236.22 ± 17.46	86.57 ± 21.87	370.22 ± 79.00	185.29 ± 38.65	612.73 ± 127.44	228.49 ± 47.72	300.90 ± 35.39	101.64 ± 5.12
		Δ change	0.00 ± 50.37	0.00 ± 61.51	-74.05 ± 122.68	146.10 ± 21.18	204.12 ± 168.20	253.82 ± 61.55	-80.50 ± 88.98	68.19 ± 27.03
		Δ change (no EpOMEs)	0.00 ± 17.46	0.00 ± 21.87	134.00 ± 79.00	98.72 ± 38.65	376.51 ± 127.44	141.92 ± 47.72	64.68 ± 35.39	15.07 ± 5.12

Values are mean ± SEM of 5-7 mice/group. ND = Below limits of detection of the assay in ≥ 50% of samples. Remaining missing values were replaced with half the minimum positive value in the original data set.

Relative concentration

Low High

Supplemental Table 1D: Specialized pro-resolving mediator (SPM) concentration (pmol/g) in young and aged mouse tibialis anterior (TA) muscle homogenates

			Uninjured		Day 1 post-injury		Day 3 post-injury		Day 5 post-injury	
Pathway	Substrate	Analyte	Young	Aged	Young	Aged	Young	Aged	Young	Aged
Specialized pro-resolving mediators	20:4n-6	LXA4	0.38 ± 0.11	0.21 ± 0.09	0.37 ± 0.16	0.31 ± 0.12	1.56 ± 0.34	0.50 ± 0.21	0.86 ± 0.21	0.43 ± 0.19
	20:4n-6	LXA5	ND	ND	ND	ND	ND	ND	ND	ND
	20:4n-6	LXB4	ND	ND	ND	ND	ND	ND	ND	ND
	20:4n-6	15-epi LXA4	ND	ND	ND	ND	ND	ND	ND	ND
	20:4n-6	15-oxo LXA4	ND	ND	ND	ND	ND	ND	ND	ND
	20:5n-3	RvE1	ND	ND	ND	ND	ND	ND	ND	ND
	20:5n-3	RvE2	ND	ND	ND	ND	ND	ND	ND	ND
	20:5n-3	RvE3	0.72 ± 0.29	ND	ND	ND	ND	ND	ND	ND
	22:5n-3	RvD5$_{(n-3DPA)}$	ND	ND	ND	ND	ND	ND	ND	ND
	22:5n-3	PD1$_{(n-3, DPA)}$	ND	ND	ND	ND	ND	ND	ND	ND
	22:5n-3	MaR1$_{(n-3DPA)}$	1.41 ± 0.48	1.47 ± 0.32	2.23 ± 1.19	1.41 ± 0.50	3.27 ± 1.00	2.51 ± 0.74	1.96 ± 0.92	ND
	22:6n-3	RvD1	ND	ND	ND	ND	ND	ND	ND	ND
	22:6n-3	8-oxoRvD1	0.51 ± 0.21	ND	0.61 ± 0.28	ND	ND	ND	ND	ND
	22:6n-3	17-oxoRvD1	ND	ND	ND	ND	ND	ND	ND	ND
	22:6n-3	RvD2	ND	ND	ND	ND	ND	ND	ND	ND
	22:6n-3	RvD3	ND	ND	ND	ND	ND	ND	ND	ND
	22:6n-3	AT-RvD3	ND	ND	ND	ND	ND	ND	ND	ND
	22:6n-3	RvD4	ND	ND	ND	ND	ND	ND	ND	ND
	22:6n-3	RvD5	ND	ND	ND	ND	ND	ND	ND	ND
	22:6n-3	RvD6	1.05 ± 0.08	1.08 ± 0.13	3.34 ± 0.84	2.86 ± 0.63	8.08 ± 1.21	3.58 ± 0.62	2.74 ± 0.33	1.95 ± 0.37
	22:6n-3	PD1	ND	ND	ND	ND	24.79 ± 10.23	ND	8.65 ± 3.15	ND
	22:6n-3	PDX	ND	ND	ND	ND	35.70 ± 15.71	ND	16.86 ± 6.19	ND
	22:6n-3	AT-PD1	ND	ND	ND	ND	ND	ND	ND	ND
	22:6n-3	22-OH-PD1	ND	ND	ND	ND	ND	ND	ND	ND
	22:6n-3	MaR1	11.21 ± 3.99	1.63 ± 0.92	ND	ND	15.21 ± 9.77	ND	7.78 ± 2.24	ND
	22:6n-3	7(S)-MaR1	ND	ND	ND	ND	ND	ND	ND	ND
	Sum		16.87 ± 4.26	5.56 ± 1.38	15.66 ± 5.93	10.64 ± 4.93	89.96 ± 34.38	9.73 ± 1.65	39.84 ± 6.47	3.13 ± 0.53
	Δ change		0.00 ± 4.26	0.00 ± 1.38	-1.21 ± 5.93	5.09 ± 4.93	73.09 ± 34.38	4.17 ± 1.65	22.97 ± 6.47	-2.42 ± 0.53

Values are mean ± SEM of 5-7 mice/group. ND = Below limits of detection of the assay in ≥ 50% of samples. Remaining missing values were replaced with half the minimum positive value in the original data set.

Relative concentration

Low High

Supplemental Table 1E: Non-enzymatic metabolite concentration (pmol/g) in young and aged mouse tibialis anterior (TA) muscle homogenates

			Uninjured		Day 1 post-injury		Day 3 post-injury		Day 5 post-injury	
Pathway	Substrate	Analyte	Young	Aged	Young	Aged	Young	Aged	Young	Aged
Non-enzymatic	20:4n-6	9-HETE	ND	ND	ND	ND	ND	ND	ND	ND
	20:4n-6	11-HETE	81.00 ± 4.17	49.13 ± 10.82	98.10 ± 13.23	110.00 ± 24.49	372.10 ± 45.42	245.83 ± 56.58	349.76 ± 22.13	211.98 ± 27.31
	20:5n-3	9-HEPE	32.30 ± 6.38	16.13 ± 4.90	15.67 ± 2.95	12.35 ± 1.89	73.42 ± 11.53	52.06 ± 8.14	81.33 ± 11.29	39.48 ± 6.62
	20:5n-3	11-HEPE	4.55 ± 0.32	2.87 ± 0.75	5.66 ± 0.85	5.96 ± 1.28	26.98 ± 3.66	14.40 ± 2.95	25.22 ± 0.76	7.95 ± 1.92
	22:6n-3	8-HDoHE	16.25 ± 1.18	9.70 ± 2.35	60.75 ± 15.07	46.36 ± 19.21	103.93 ± 18.59	34.33 ± 7.45	39.90 ± 4.62	15.16 ± 1.52
	22:6n-3	10-HDoHE	23.32 ± 3.20	14.40 ± 3.45	58.36 ± 12.35	47.81 ± 17.75	107.11 ± 13.75	44.49 ± 7.03	50.35 ± 3.61	23.45 ± 2.66
	22:6n-3	11-HDoHE	10.77 ± 3.62	7.58 ± 1.44	41.30 ± 10.50	32.79 ± 13.25	70.76 ± 12.86	29.54 ± 5.39	31.08 ± 3.62	14.51 ± 1.47
	22:6n-3	13-HDoHE	18.29 ± 0.79	14.13 ± 2.88	58.98 ± 12.83	47.74 ± 16.51	121.57 ± 15.46	53.97 ± 10.16	64.41 ± 5.48	30.42 ± 3.38
	22:6n-3	16-HDoHE	25.82 ± 1.91	19.45 ± 4.00	83.24 ± 19.54	71.69 ± 28.18	159.39 ± 24.12	61.79 ± 9.86	70.10 ± 9.91	30.70 ± 3.76
	22:6n-3	20-HDoHE	23.50 ± 0.98	18.28 ± 3.47	70.68 ± 15.95	59.62 ± 17.82	154.73 ± 21.60	67.20 ± 10.48	66.77 ± 5.18	35.17 ± 3.62
	Sum		235.83 ± 9.32	151.70 ± 31.01	492.78 ± 97.68	434.35 ± 136.70	1190.03 ± 150.77	603.64 ± 114.54	778.96 ± 40.40	408.86 ± 46.02
	Δ change		0.00 ± 9.32	0.00 ± 31.01	256.95 ± 97.68	282.64 ± 136.70	954.20 ± 150.77	451.94 ± 114.54	543.13 ± 40.40	257.15 ± 46.02

Values are mean ± SEM of 5-7 mice/group. ND = Below limits of detection of the assay in ≥ 50% of samples. Remaining missing values were replaced with half the minimum positive value in the original data set.

Relative concentration

Low High
