# Supplementary material for: Metabolipidomic profiling reveals an age‐related deficiency of skeletal muscle pro‐resolving mediators that contributes to maladaptive tissue remodeling
Source: Aging Cell. 2021 Jun 2;20(6):e13393. doi: 10.1111/acel.13393 (PMC8208786; doi:10.1111/acel.13393)
Supplement: Supplementary file 3 — Table S2 [file ACEL-20-e13393-s001.pdf]

Supplemental Table 2A: Aged vs. young mouse tibialis anterior (TA) muscle lipid mediators - Uninjured

Row no.	Analyte	Fold change	Log2 fold change	P.Value	BH FDR
1	20-HETE	0.28596	-1.8061	0.00024485	0.01290268
2	5(6)-EpETrE	0.17183	-2.541	0.00038586	0.01290268
3	5(S)-HETrE	0.46477	-1.1054	0.00039498	0.01290268
4	9(S)-HOTrE	0.39205	-1.3509	0.00057999	0.01420976
5	11(12)-EpETrE	0.2623	-1.9307	0.00082381	0.01614668
6	14(15)-EpETrE	0.18781	-2.4127	0.00174660	0.02852780
7	15-HETE	0.56338	-0.82782	0.00227610	0.03186540
8	12(13)-EpOME	0.45753	-1.128	0.00354470	0.03917773
9	8-HETE	0.44239	-1.1766	0.00379700	0.03917773
10	9(10)-EpOME	0.24421	-2.0338	0.00411900	0.03917773
11	11-HETE	0.6285	-0.67002	0.00439750	0.03917773
12	19(20)-EpDPE	0.51761	-0.95005	0.00548390	0.04233977
13	9-OxoOTrE	0.43589	-1.1979	0.00561650	0.04233977
14	15(S)-HEDE	0.59013	-0.76089	0.00659000	0.04326961
15	10(11)-EpDPE	0.42096	-1.2482	0.00662290	0.04326961
16	13-HODE	0.63035	-0.66578	0.00812260	0.04375522
17	13-OxoODE	0.62418	-0.67996	0.00817750	0.04375522
18	9-HODE	0.66817	-0.58171	0.00866830	0.04375522
19	7(8)-EpDPE	0.44952	-1.1535	0.00969240	0.04375522
20	8(S)-HETrE	0.42651	-1.2293	0.00975320	0.04375522
21	16(17)-EpDPE	0.47685	-1.0684	0.00980700	0.04375522
22	13(14)-EpDPE	0.4525	-1.144	0.00982260	0.04375522
23	5-HETE	0.49976	-1.0007	0.01027100	0.04376339
24	MaR1	0.17418	-2.5213	0.01151500	0.04701958
25	11(12)-EpETE	0.18412	-2.4413	0.01370400	0.05371968
26	13(S)-HOTrE	0.40047	-1.3202	0.01497100	0.05642915
27	8(S),15(S)-DiHETE	0.22283	-2.166	0.01916300	0.06955459
28	12-OxoLTB4	0.19748	-2.3403	0.02320300	0.08121050
29	RvE3	0.24793	-2.012	0.02538200	0.08375407
30	8-oxoRvD1	0.24243	-2.0444	0.02563900	0.08375407
31	8-HDoHE	0.62217	-0.68462	0.02982200	0.09427600
32	8(9)-EpETE	0.40548	-1.3023	0.03139700	0.09615331
33	11,12-DiHETrE	0.71099	-0.4921	0.03805100	0.11299994
34	5-oxoETE	0.49828	-1.005	0.03947600	0.11378376
35	8(9)-EpETrE	0.40069	-1.3194	0.04241700	0.11876760
36	7-HDoHE	0.6714	-0.57475	0.05211200	0.14186044
37	PGF1a	0.74212	-0.43028	0.05363700	0.14206557
38	11(R)-HEDE	0.75852	-0.39874	0.05695200	0.14687621
39	17(18)-EpETE	0.44174	-1.1787	0.06006200	0.15092503
40	15(S)-HEPE	0.48265	-1.0509	0.06234700	0.15275015
41	12(S)-HHTrE	0.73601	-0.4422	0.07117100	0.17011605
42	5(S),12(S)-DiHETE	0.47238	-1.082	0.08057100	0.18799900
43	8,9-DiHETrE	0.57933	-0.78753	0.08889400	0.20259563
44	12-HETE	0.5881	-0.76586	0.09125100	0.20324086
45	20-HDoHE	0.84631	-0.24074	0.09844200	0.21438480
46	12-OxoETE	0.27508	-1.8621	0.11196000	0.23718000
47	13-HDoHE	0.8384	-0.25429	0.11566000	0.23718000
48	10-HDoHE	0.70199	-0.51048	0.11745000	0.23718000
49	16-HDoHE	0.794	-0.33279	0.11859000	0.23718000
50	18-HEPE	0.70377	-0.50682	0.14039000	0.27516440
51	8-HEPE	0.69073	-0.53381	0.14584000	0.28024157
52	14-HDoHE	0.60414	-0.72704	0.17094000	0.31735358
53	TXB2	0.67288	-0.57157	0.17163000	0.31735358
54	PGF2a	0.81871	-0.28858	0.17776000	0.32106175
55	PGJ2	1.4919	0.57711	0.18175000	0.32106175

56	LXA5	2.359	1.2381	0.18647000	0.32106175
57	LXB4	2.424	1.2774	0.18674000	0.32106175
58	11-HEPE	0.74589	-0.42296	0.19491000	0.32933069
59	11dh-2,3-dinor TXB2	0.69993	-0.51472	0.20968000	0.34828203
60	9-OxoODE	0.79997	-0.32198	0.22911000	0.37119031
61	13,14dh-15k-PGD2	0.76698	-0.38274	0.23507000	0.37119031
62	14(15)-EpETE	0.34104	-1.552	0.23668000	0.37119031
63	TXB3	0.635	-0.65518	0.24231000	0.37119031
64	19,20-DiHDoPE	1.3485	0.43137	0.24241000	0.37119031
65	5(S),15(S)-DiHEPE	0.35126	-1.5094	0.25623000	0.38631600
66	9-HEPE	0.72207	-0.46979	0.26680000	0.39615758
67	12-HEPE	0.72228	-0.46938	0.27741000	0.40576388
68	14,15-DiHETrE	0.85156	-0.23181	0.29146000	0.42004529
69	PGF3a	1.1538	0.20634	0.33122000	0.47042841
70	15-keto PGE2	0.83223	-0.26494	0.36114000	0.49818028
71	5-HEPE	0.8524	-0.2304	0.36326000	0.49818028
72	MaR1(n-3DPA)	1.1899	0.25081	0.36601000	0.49818028
73	LXA4	0.63557	-0.65388	0.41384000	0.55556603
74	4-HDoHE	0.89148	-0.16572	0.44987000	0.59577378
75	7(S)-Maresin1	0.61298	-0.70608	0.45966000	0.59636868
76	D12-PGJ2	0.88311	-0.17933	0.46249000	0.59636868
77	13,14dh-15k-PGE1	0.87496	-0.19271	0.50013000	0.63652909
78	8-isoPGF2a & 11bPGF2a	0.96112	-0.057215	0.50962000	0.64029179
79	PGA2	1.8696	0.90272	0.53204000	0.65999899
80	6kPGF1a	0.9145	-0.12894	0.54526000	0.66794350
81	15-OxoETE	1.7393	0.79852	0.60206000	0.72234366
82	12,13-DiHOME	1.1148	0.15683	0.60441000	0.72234366
83	PGE3	1.4124	0.49815	0.62553000	0.73857759
84	9,10-DiHOME	0.95076	-0.072842	0.63423000	0.73993500
85	15-keto PGF2a	1.5371	0.62022	0.67683000	0.77400628
86	13(S)-HOTrE(g)	0.65525	-0.60988	0.67923000	0.77400628
87	PGD2	0.97527	-0.036126	0.69889000	0.78481483
88	5,6-DiHETrE	1.273	0.34819	0.70991000	0.78481483
89	5(S),15(S)-DiHETE	0.59039	-0.76026	0.71274000	0.78481483
90	RvD6	0.98683	-0.019133	0.75664000	0.82389689
91	13,14dh-15k-PGE2	0.99388	-0.008859	0.77891000	0.83882615
92	15-OxoEDE	0.8035	-0.31563	0.82435000	0.87255828
93	19(R)-OH PGF2a & 20-OH PGF2a	0.95429	-0.067497	0.82804000	0.87255828
94	LTB5	0.8488	-0.2365	0.84866000	0.88477319
95	17-HDoHE	0.80591	-0.3113	0.87536000	0.90300295
96	tetranor 12-HETE	1.0446	0.063008	0.88996000	0.90850083
97	PGE2	1.0461	0.065012	0.98855000	0.99874124
98	11-HDoHE	0.73214	-0.4498	0.99910000	0.99910000

Supplemental Table 2B: Aged vs. young mouse tibialis anterior (TA) muscle lipid mediators - Day 1 post-injury

Row no.	Analyte	Fold change	Log2 fold change	P.Value	BH FDR
1	8-isoPGF2a & 11bPGF2a	2.7424	1.4554	0.00631390	0.55685600
2	9-HODE	1.7047	0.76953	0.01184800	0.55685600
3	19(R)-OH PGF2a & 20-OH PGF2a	0.57859	-0.78938	0.02212500	0.69325000
4	12(S)-HHTrE	1.3909	0.47607	0.03550300	0.83432050
5	PGF2a	1.3957	0.48096	0.05411300	0.85040457
6	PGF1a	2.9443	1.5579	0.06086300	0.85040457
7	13-HODE	1.5239	0.60778	0.06332800	0.85040457
8	8-oxoRvD1	0.29651	-1.7538	0.07922000	0.88963689
9	11,12-DiHETrE	1.2338	0.30307	0.08517800	0.88963689
10	9(10)-EpOME	0.79344	-0.3338	0.09583500	0.89094909
11	13-OxoODE	1.7097	0.77374	0.10426000	0.89094909
12	5(6)-EpETrE	0.5719	-0.80616	0.13925000	0.95282429
13	11dh-2,3-dinor TXB2	0.38969	-1.3596	0.18636000	0.95282429
14	TXB3	0.42259	-1.2427	0.19320000	0.95282429
15	17(18)-EpETE	0.75547	-0.40455	0.22325000	0.95282429
16	9-OxoOTrE	1.6601	0.73126	0.23319000	0.95282429
17	19(20)-EpDPE	0.52715	-0.92371	0.26750000	0.95282429
18	11(R)-HEDE	1.2677	0.34221	0.27109000	0.95282429
19	13(S)-HOTrE(g)	2.4259	1.2785	0.28362000	0.95282429
20	15-OxoEDE	1.4479	0.53401	0.30823000	0.95282429
21	10(11)-EpDPE	0.6357	-0.65359	0.33039000	0.95282429
22	16(17)-EpDPE	0.61256	-0.70708	0.33679000	0.95282429
23	19,20-DiHDoPE	0.34039	-1.5548	0.34387000	0.95282429
24	9-OxoODE	1.3899	0.47494	0.35091000	0.95282429
25	14(15)-EpETrE	0.77021	-0.37668	0.35186000	0.95282429
26	13(14)-EpDPE	0.60901	-0.71545	0.36428000	0.95282429
27	8(9)-EpETrE	1.2473	0.31877	0.36529000	0.95282429
28	14-HDoHE	0.69165	-0.53188	0.37486000	0.95282429
29	7(8)-EpDPE	0.64503	-0.63256	0.39121000	0.95282429
30	8(9)-EpETE	0.76064	-0.39472	0.39854000	0.95282429
31	9(S)-HOTrE	1.3092	0.38865	0.41367000	0.95282429
32	PGJ2	0.88526	-0.17582	0.41952000	0.95282429
33	8-HEPE	0.79075	-0.3387	0.41963000	0.95282429
34	9-HEPE	0.78828	-0.34321	0.44618000	0.95282429
35	PGE1	0.65011	-0.62125	0.45959000	0.95282429
36	15(S)-HEPE	0.77742	-0.36323	0.48199000	0.95282429
37	PGD2	1.1212	0.16501	0.48499000	0.95282429
38	5(S),15(S)-DiHEPE	0.48427	-1.0461	0.49775000	0.95282429
39	tetranor 12-HETE	1.3403	0.42253	0.50513000	0.95282429
40	13,14dh-15k-PGE2	0.46581	-1.1022	0.52803000	0.95282429
41	8-HDoHE	0.76309	-0.39008	0.53151000	0.95282429
42	12-OxoLTB4	1.0238	0.033887	0.53436000	0.95282429
43	PGA2	1.2112	0.27646	0.54920000	0.95282429
44	11-HDoHE	0.79396	-0.33285	0.55118000	0.95282429
45	10-HDoHE	0.81921	-0.2877	0.55970000	0.95282429
46	13(S)-HOTrE	1.2193	0.28602	0.56082000	0.95282429
47	5-oxoETE	1.142	0.19159	0.59986000	0.95282429
48	12(13)-EpOME	1.2518	0.32406	0.60005000	0.95282429
49	13,14dh-15k-PGD2	0.65004	-0.6214	0.61081000	0.95282429
50	11(12)-EpETE	0.65584	-0.60859	0.61590000	0.95282429
51	12-HETE	1.0292	0.041456	0.61760000	0.95282429
52	20-HETE	0.95116	-0.072246	0.62338000	0.95282429
53	15-HETE	1.143	0.19285	0.62351000	0.95282429
54	5(S)-HETrE	1.0716	0.099816	0.62649000	0.95282429
55	16-HDoHE	0.8612	-0.21558	0.63348000	0.95282429

56	13-HDoHE	0.80937	-0.30514	0.64046000	0.95282429
57	8(S)-HETrE	0.8444	-0.244	0.64109000	0.95282429
58	7-HDoHE	0.85341	-0.22868	0.64740000	0.95282429
59	PGE2	1.0444	0.062623	0.66606000	0.95282429
60	15-keto PGE2	0.9032	-0.14688	0.67154000	0.95282429
61	13,14dh-15k-PGE1	1.6859	0.7535	0.67242000	0.95282429
62	5(S),12(S)-DiHETE	0.8603	-0.21708	0.67405000	0.95282429
63	8,9-DiHETrE	1.0353	0.049996	0.67957000	0.95282429
64	20-HDoHE	0.84356	-0.24543	0.69663000	0.95282429
65	MaR1	0.28449	-1.8136	0.69939000	0.95282429
66	11-HETE	1.1212	0.16506	0.69991000	0.95282429
67	Bicyclo PGE1	0.77226	-0.37283	0.70018000	0.95282429
68	11(12)-EpETrE	0.91978	-0.12063	0.70035000	0.95282429
69	15-OxoETE	1.1157	0.15791	0.70316000	0.95282429
70	D12-PGJ2	1.0394	0.055768	0.70955000	0.95282429
71	14,15-DiHETrE	0.9425	-0.085429	0.74620000	0.97616473
72	12-HEPE	0.87001	-0.2009	0.75341000	0.97616473
73	18-HEPE	0.91049	-0.13529	0.77486000	0.97616473
74	4-HDoHE	0.74806	-0.41878	0.77801000	0.97616473
75	TXB2	0.98027	-0.028752	0.79354000	0.97616473
76	5-HETE	1.0508	0.071549	0.79573000	0.97616473
77	AT-PD1	1.0212	0.030313	0.81062000	0.97616473
78	PGE3	1.0636	0.088944	0.84350000	0.97616473
79	6kPGF1a	0.97489	-0.036692	0.85854000	0.97616473
80	11-HEPE	1.0527	0.074046	0.87317000	0.97616473
81	15(S)-HEDE	1.1471	0.19798	0.88451000	0.97616473
82	12,13-DiHOME	0.96168	-0.056373	0.88815000	0.97616473
83	RvD6	0.85684	-0.22291	0.89527000	0.97616473
84	8-HETE	1.1624	0.21712	0.89824000	0.97616473
85	LTB5	0.78002	-0.35841	0.91673000	0.97616473
86	5,6-DiHETrE	1.2516	0.32378	0.91680000	0.97616473
87	17-HDoHE	1.0425	0.060026	0.92219000	0.97616473
88	9,10-DiHOME	1.0054	0.0077581	0.94273000	0.97616473
89	14(15)-EpETE	1.2421	0.31279	0.94547000	0.97616473
90	5-HEPE	0.94535	-0.081077	0.95055000	0.97616473
91	PD1	1.0087	0.01249	0.95063000	0.97616473
92	MaR1(n-3DPA)	0.63881	-0.64654	0.96086000	0.97616473
93	LXA4	0.83625	-0.25799	0.96578000	0.97616473
94	15-keto PGF2a	0.96467	-0.051889	0.97901000	0.97901000

Supplemental Table 2C: Aged vs. young mouse tibialis anterior (TA) muscle lipid mediators - Day 3 post-injury

Row no.	Analyte	Fold change	Log2 fold change	P.Value	BH FDR
1	8(S)-HETrE	0.33121	-1.5942	0.00121530	0.02985713
2	10-HDoHE	0.41537	-1.2675	0.00141830	0.02985713
3	16(17)-EpDPE	0.30258	-1.7246	0.00160240	0.02985713
4	16-HDoHE	0.38768	-1.367	0.00186170	0.02985713
5	8-HDoHE	0.33033	-1.598	0.00213620	0.02985713
6	PGE1	0.25559	-1.9681	0.00255470	0.02985713
7	20-HDoHE	0.43429	-1.2033	0.00258700	0.02985713
8	13,14dh-15k-PGD2	0.3596	-1.4755	0.00287400	0.02985713
9	10(11)-EpDPE	0.26475	-1.9173	0.00333650	0.02985713
10	7-HDoHE	0.42209	-1.2444	0.00336150	0.02985713
11	11(12)-EpETE	0.19632	-2.3487	0.00338630	0.02985713
12	13-HDoHE	0.44393	-1.1716	0.00347850	0.02985713
13	RvD6	0.44357	-1.1728	0.00379540	0.03007125
14	15(S)-HEDE	0.44399	-1.1714	0.00412730	0.03036514
15	5(6)-EpETrE	0.25901	-1.9489	0.00463420	0.03181992
16	PD1	0.091698	-3.447	0.00494290	0.03181992
17	12(13)-EpOME	0.50813	-0.97673	0.00567840	0.03440442
18	17-HDoHE	0.41319	-1.2751	0.00693760	0.03809970
19	4-HDoHE	0.36217	-1.4653	0.00702810	0.03809970
20	7(8)-EpDPE	0.29835	-1.7449	0.00842570	0.03974208
21	14-HDoHE	0.44788	-1.1588	0.00848020	0.03974208
22	11-HDoHE	0.41751	-1.2601	0.00848860	0.03974208
23	11(R)-HEDE	0.50216	-0.99378	0.00987970	0.04424387
24	PGF3a	2.001	1.0007	0.01117000	0.04767664
25	14(15)-EpETrE	0.35106	-1.5102	0.01157200	0.04767664
26	20-HETE	0.31611	-1.6615	0.01229100	0.04869127
27	5-HEPE	0.44125	-1.1803	0.01318800	0.05030978
28	18-HEPE	0.52124	-0.93999	0.01384700	0.05093718
29	13(14)-EpDPE	0.35696	-1.4862	0.01445600	0.05134372
30	11(12)-EpETrE	0.38225	-1.3874	0.01554000	0.05164806
31	15-keto PGE2	0.51188	-0.96613	0.01586400	0.05164806
32	5-HETE	0.44971	-1.1529	0.01604600	0.05164806
33	5-oxoETE	0.41227	-1.2783	0.01691400	0.05279218
34	PGE3	0.53218	-0.91001	0.01754300	0.05314497
35	PDX	0.13267	-2.914	0.02003600	0.05896309
36	Bicyclo PGE2	0.46215	-1.1136	0.02082300	0.05957692
37	PGJ2	0.55578	-0.84743	0.02147800	0.05979011
38	LXA4	0.32225	-1.6337	0.02540000	0.06869610
39	19,20-DiHDoPE	0.62639	-0.67488	0.02681300	0.06869610
40	13-OxoODE	0.58712	-0.76828	0.02732300	0.06869610
41	15(S)-HEPE	0.4619	-1.1144	0.02794600	0.06869610
42	11-HEPE	0.53369	-0.90594	0.02801200	0.06869610
43	17(18)-EpETE	0.4324	-1.2096	0.02900100	0.06946751
44	15-keto PGF2a	0.51098	-0.96866	0.03281000	0.07680523
45	8-HEPE	0.5567	-0.84504	0.03392300	0.07764598
46	9-OxoODE	0.58748	-0.76739	0.03647400	0.08167004
47	19(20)-EpDPE	0.55437	-0.85108	0.03978900	0.08449994
48	PGD2	0.51608	-0.95434	0.04018900	0.08449994
49	PGD3	0.32871	-1.6051	0.04019900	0.08449994
50	5(S)-HETrE	0.50552	-0.98416	0.04159000	0.08567540
51	6kPGF1a	0.66691	-0.58444	0.05120000	0.10194425
52	8(9)-EpETE	0.30805	-1.6987	0.05146700	0.10194425
53	5,6-DiHETE(EPA)	0.319	-1.6484	0.05791000	0.11159207
54	15-HETE	0.61905	-0.69188	0.05861800	0.11159207
55	15(R)-PGE1	4.0322	2.0116	0.05958800	0.11159207

56	8(S),15(S)-DiHETE	3.4417	1.7831	0.06128400	0.11271879
57	14(15)-EpETE	0.52705	-0.92399	0.06668500	0.11914436
58	8-HETE	0.56862	-0.81446	0.06709100	0.11914436
59	15-OxoEDE	0.47313	-1.0797	0.07055800	0.12317753
60	13,14dh-15k-PGE2	0.68613	-0.54345	0.08048500	0.13816592
61	11-HETE	0.66065	-0.59805	0.08695900	0.14683241
62	8-isoPGF2a & 11bPGF2a	1.6194	0.69545	0.09736000	0.16174323
63	13-HODE	0.75296	-0.40935	0.10119000	0.16543762
64	PGE2	0.72766	-0.45867	0.10798000	0.17378031
65	LTB5	0.24837	-2.0094	0.11443000	0.18132754
66	8(9)-EpETrE	1.485	0.57044	0.11811000	0.18432318
67	9-HODE	0.76456	-0.3873	0.14548000	0.22364836
68	11dh-2,3-dinor TXB2	0.43739	-1.193	0.14804000	0.22423706
69	MaR1	0.18687	-2.4199	0.17109000	0.25345357
70	9-OxoOTrE	0.62881	-0.66931	0.17225000	0.25345357
71	9(S)-HOTrE	0.74063	-0.43317	0.19716000	0.28602085
72	15d-D12,14-PGJ2	0.43339	-1.2063	0.21540000	0.30814167
73	Bicyclo PGE1	1.7597	0.8153	0.25679000	0.36232014
74	19(R)-OH PGF2a & 20-OH PGF2a	0.66223	-0.59461	0.30194000	0.42026784
75	9-HEPE	0.70906	-0.49602	0.32040000	0.43493105
76	PGF2a	1.2196	0.28636	0.32092000	0.43493105
77	AT-PD1	0.28716	-1.8001	0.35212000	0.47101766
78	13(S)-HOTrE	0.81521	-0.29475	0.38832000	0.51278154
79	11,12-DiHETrE	0.84146	-0.24903	0.40314000	0.52561291
80	8,9-DiHETrE	0.63932	-0.64539	0.41335000	0.53218813
81	12-OxoLTB4	0.62306	-0.68256	0.42945000	0.54609074
82	8-oxoRvD1	0.47064	-1.0873	0.43655000	0.54834939
83	tetranor 12-HETE	0.86775	-0.20466	0.44532000	0.55262602
84	12-HEPE	0.75435	-0.40669	0.45555000	0.55859107
85	13,14dh-15k-PGE1	1.2576	0.33068	0.49505000	0.59988412
86	12,13-DiHOME	1.118	0.16095	0.53568000	0.64157023
87	15-OxoETE	0.60351	-0.72855	0.64795000	0.76711322
88	14,15-DiHETrE	0.87947	-0.18529	0.66390000	0.77706477
89	LXA5	0.53442	-0.90396	0.70977000	0.82141921
90	12-HETE	0.86768	-0.20476	0.72406000	0.82842228
91	PGA2	0.71254	-0.48897	0.73879000	0.82842228
92	12(S)-HHTrE	1.1118	0.15294	0.73995000	0.82842228
93	13(S)-HOTrE(g)	1.3989	0.48431	0.75529000	0.83461777
94	LXB4	0.58761	-0.76708	0.76169000	0.83461777
94	MaR1(n-3DPA)	0.77211	-0.37312	0.77013000	0.83498305
94	TXB3	0.60665	-0.72106	0.79893000	0.85718531
94	TXB2	0.93776	-0.092706	0.81805000	0.86865103
94	5(S),12(S)-DiHETE	0.84246	-0.24731	0.85084000	0.88857386
94	9(10)-EpOME	0.63361	-0.65833	0.86225000	0.88857386
94	D12-PGJ2	0.61573	-0.69962	0.86455000	0.88857386
94	5,6-DiHETrE	1.1706	0.22719	0.87132000	0.88857386
94	9,10-DiHOME	1.0197	0.028105	0.94150000	0.95073039
94	PGF1a	0.83859	-0.25396	0.95783000	0.95783000

Supplemental Table 2D: Aged vs. young mouse tibialis anterior (TA) muscle lipid mediators - Day 5 post-injury

Row no.	Analyte	Fold change	Log2 fold change	P.Value	BH FDR
1	16(17)-EpDPE	0.26979	-1.8901	0.00000574	0.00023100
2	5-HEPE	0.28822	-1.7947	0.00000846	0.00023100
3	11(12)-EpETrE	0.26594	-1.9109	0.00000998	0.00023100
4	5-HETE	0.42911	-1.2206	0.00001404	0.00023100
5	7(8)-EpDPE	0.23279	-2.1029	0.00001479	0.00023100
6	10(11)-EpDPE	0.20672	-2.2743	0.00001622	0.00023100
7	19(20)-EpDPE	0.44222	-1.1772	0.00001655	0.00023100
8	15(S)-HEDE	0.50389	-0.98883	0.00005355	0.00065660
9	4-HDoHE	0.42835	-1.2231	0.00007540	0.00082102
10	8-HDoHE	0.38006	-1.3957	0.00010390	0.00096245
11	15(S)-HEPE	0.19167	-2.3833	0.00010803	0.00096245
12	9(S)-HOTrE	0.41286	-1.2763	0.00013638	0.00105900
13	10-HDoHE	0.46576	-1.1023	0.00014048	0.00105900
14	13-HDoHE	0.47228	-1.0823	0.00017159	0.00112399
15	5(S)-HETrE	0.47595	-1.0711	0.00017204	0.00112399
16	20-HETE	0.10741	-3.2189	0.00020467	0.00124523
17	8(S)-HETrE	0.40891	-1.2901	0.00021601	0.00124523
18	20-HDoHE	0.5267	-0.92496	0.00027904	0.00149472
19	13(14)-EpDPE	0.28459	-1.813	0.00030122	0.00149472
20	8-HEPE	0.33375	-1.5831	0.00032162	0.00149472
21	11-HEPE	0.31527	-1.6653	0.00032713	0.00149472
22	11-HDoHE	0.46688	-1.0989	0.00033555	0.00149472
23	5(6)-EpETrE	0.37254	-1.4245	0.00047254	0.00193085
24	18-HEPE	0.33812	-1.5644	0.00047286	0.00193085
25	12(13)-EpOME	0.46065	-1.1182	0.00050028	0.00196110
26	11(R)-HEDE	0.50631	-0.98192	0.00063620	0.00239798
27	16-HDoHE	0.43796	-1.1911	0.00069258	0.00251381
28	17(18)-EpETE	0.22254	-2.1679	0.00115570	0.00404495
29	5-oxoETE	0.49626	-1.0108	0.00122180	0.00412884
30	7-HDoHE	0.41241	-1.2779	0.00161140	0.00526391
31	13(S)-HOTrE	0.58657	-0.76961	0.00190890	0.00603459
32	8-HETE	0.49303	-1.0203	0.00203430	0.00623004
33	11-HETE	0.60606	-0.72246	0.00275050	0.00816815
34	14-HDoHE	0.59075	-0.75937	0.00340060	0.00980173
35	9-OxoOTrE	0.48028	-1.0581	0.00500580	0.01399032
36	PGE1	0.29376	-1.7673	0.00513930	0.01399032
37	17-HDoHE	0.41166	-1.2805	0.00619390	0.01640546
38	15-HETE	0.66712	-0.58399	0.00689370	0.01777849
39	MaR1	0.1752	-2.5129	0.01076000	0.02703795
40	9-HEPE	0.48543	-1.0427	0.01130900	0.02770705
41	13-HODE	0.81014	-0.30375	0.01367200	0.03267941
42	12-HEPE	0.51286	-0.96336	0.01876600	0.04378733
43	15-OxoETE	0.59896	-0.73948	0.02004800	0.04569079
44	13,14dh-15k-PGD2	0.37953	-1.3977	0.02133600	0.04752109
45	tetranor 12-HETE	0.49984	-1.0005	0.02444000	0.05322489
46	6kPGF1a	0.55275	-0.85531	0.02759300	0.05878509
47	PGD2	0.4944	-1.0162	0.03156800	0.06582264
48	PGD3	0.29607	-1.756	0.03423600	0.06989850
49	19,20-DiHDoPE	0.73493	-0.44432	0.04047500	0.08095000
50	PD1	0.26857	-1.8966	0.04176200	0.08112478
51	PDX	0.22733	-2.1371	0.04221800	0.08112478
52	8(S),15(S)-DiHETE	0.23518	-2.0882	0.04469300	0.08422912
53	9,10-DiHOME	0.77375	-0.37006	0.04806500	0.08887491
54	8(9)-EpETE	0.089799	-3.4771	0.06129400	0.10921655
55	15-keto PGF2a	0.54926	-0.86443	0.06129500	0.10921655

56	15-OxoEDE	0.34604	-1.531	0.06479900	0.11339825
57	PGJ2	0.4954	-1.0133	0.08812900	0.15152004
58	PGE3	0.50816	-0.97665	0.10565000	0.17851207
59	MaR1(n-3DPA)	0.30634	-1.7068	0.10945000	0.18179831
60	12,13-DiHOME	0.7579	-0.39992	0.11346000	0.18531800
61	15-keto PGE2	0.64735	-0.62739	0.14840000	0.23841311
62	RvD6	0.71279	-0.48846	0.15832000	0.25024774
63	9(10)-EpOME	0.39691	-1.3331	0.16484000	0.25641778
64	TXB3	0.34203	-1.5478	0.17278000	0.26456938
65	13-OxoODE	0.77082	-0.37554	0.17574000	0.26496185
66	13,14dh-15k-PGE2	1.4302	0.51622	0.19829000	0.29443061
67	11dh-TXB2	0.42963	-1.2188	0.22709000	0.32425217
68	LXA4	0.52856	-0.91986	0.22779000	0.32425217
69	11dh-2,3-dinor TXB2	0.41344	-1.2743	0.22830000	0.32425217
70	12-OxoLTB4	0.4256	-1.2324	0.24789000	0.34704600
71	8,9-DiHETrE	0.65638	-0.6074	0.28507000	0.39347690
72	13,14dh-15k-PGE1	1.4366	0.52261	0.30017000	0.40856472
73	D12-PGJ2	0.39985	-1.3225	0.30762000	0.41296932
74	PGF2a	1.2337	0.30304	0.33183000	0.43945054
75	PGF1a	1.2026	0.26622	0.34171000	0.44650107
76	19(R)-OH PGF2a & 20-OH PGF2a	0.6217	-0.6857	0.41491000	0.53501553
77	15(R)-PGE1	1.5291	0.61271	0.42269000	0.53796909
78	TXB2	1.1313	0.17793	0.43829000	0.55067205
79	11,12-DiHETrE	0.88349	-0.17871	0.48848000	0.60230800
80	11(12)-EpETE	0.25832	-1.9527	0.49168000	0.60230800
81	13(S)-HOTrE(g)	1.2368	0.30661	0.51871000	0.62757506
82	PGE2	0.84865	-0.23675	0.55178000	0.65424800
83	8-isoPGF2a & 11bPGF2a	1.2246	0.29232	0.56268000	0.65424800
84	9-HODE	1.0838	0.11604	0.56633000	0.65424800
85	12-HETE	0.86069	-0.21644	0.56746000	0.65424800
86	LTB5	0.40975	-1.2872	0.58339000	0.66479326
87	14(15)-EpETrE	0.87452	-0.19344	0.62718000	0.70647862
88	5,6-DiHETrE	1.195	0.25701	0.65531000	0.72314413
89	PGF3a	0.55904	-0.83898	0.66220000	0.72314413
90	14(15)-EpETE	0.5795	-0.78711	0.66722000	0.72314413
91	PGA2	0.57751	-0.79208	0.67525000	0.72314413
92	9-OxoODE	0.88739	-0.17236	0.67887000	0.72314413
93	5(S),12(S)-DiHETE	0.91592	-0.12671	0.68640000	0.72330323
94	5(S),15(S)-DiHEPE	1.0974	0.13413	0.69455000	0.72410532
94	12(S)-HHTrE	1.1183	0.1613	0.71500000	0.73757895
94	Bicyclo PGE2	0.68856	-0.53834	0.74450000	0.76001042
94	14,15-DiHETrE	0.95096	-0.072537	0.81623000	0.82464474
94	8(9)-EpETrE	1.0188	0.026862	0.97746000	0.97746000
