# Supplementary material for: Metabolipidomic profiling reveals an age‐related deficiency of skeletal muscle pro‐resolving mediators that contributes to maladaptive tissue remodeling
Source: Aging Cell. 2021 Jun 2;20(6):e13393. doi: 10.1111/acel.13393 (PMC8208786; doi:10.1111/acel.13393)
Supplement: Supplementary file 4 — Supplementary Material [file ACEL-20-e13393-s003.docx]

**Supporting Information:**

**Supplemental Experimental Procedures:**

***Resolvin D1 handling:*** Single use aliquots of RvD1 were prepared in amber glass vials (ThermoFisher, C4010-88AW) which were purged with nitrogen gas and stored at -80°C. On the day of use, the ethanol was evaporated to dryness under a gentle stream of nitrogen gas and RvD1 was re-suspended in sterile saline containing 0.1% ethanol. RvD1 stocks were handled in a darkroom and aqueous solutions of RvD1 were protected from light and used within 30 minutes of preparation.

***Muscle Tissue Collection:*** Animals were euthanized via induction of bilateral pneumothorax while under deep isoflurane anesthesia. Muscles were rapidly dissected, blotted dry, weighed, and snap frozen in liquid nitrogen. Muscles for histological analysis were cut transversely at the mid-belly, oriented longitudinally on a plastic support, covered with a thin layer of optimal cutting temperature (OCT) compound, and rapidly frozen in isopentane cooled on liquid nitrogen. Samples were stored at -80°C until analysis.

***Immunohistochemistry Antibodies:*** Primary antibodies used included embryonic myosin heavy chain (DSHB, F1.652s, 1:20), Pax7 (DSHB, Pax7c, 1:100), myosin heavy chain types I (DSHB, BA-D5c, 1:100), IIa (DSHB, SC-71c, 1:100), IIb (DSHB, BF-F3c, 1:100), laminin (Abcam, ab7463, 1:200), rat anti-mouse Ly-6G (Gr-1) (BD Biosciences, BD550291, 1:50), rat anti-mouse CD68 (Bio-Rad, MCA1957, 1:50), and rabbit polyclonal CD163 (Santa Cruz, sc-33560, 1:50). Antibody binding was visualized with standard Alexa Fluor conjugated secondary antibodies (Invitrogen, 1:500 in PBS) except for Pax7 which was detected using a Tyramide SuperBoost Kit (Invitrogen, B40913). Fluorescent dyes including 4′,6-diamidino-2-phenylindole (DAPI, Invitrogen, D21490, 2 µg/mL), wheat germ agglutinin (WGA) Alexa Fluor 647 conjugate (Invitrogen, W32466, 5 µg/mL), WGA CF405S conjugate (Biotium, 29027, 100 µg/mL), and phalloidin (Invitrogen, ActinRed 555 ReadyProbes, R37112) were used to counterstain cell nuclei, extracellular matrix, and muscle fibers respectively.

***Flow Cytometry Analysis of Basal Muscle Inflammation:*** The entire hind-limb musculature from each mouse was dissected, pooled, finely minced, and digested for 30 min at 37°C in Dulbecco's Modified Eagle's Medium (DMEM, Gibco, 11995-073) supplemented with 10 mg/mL collagenase II (ThermoFisher) and 20 mg/mL dispase (Sigma-Aldrich). The resulting digest solution was deactivated by adding fetal bovine serum (FBS) and then filtered through a 70-μm cell strainer and centrifuged at 450 x g for 5 min. Cells were Fc blocked with a CD16/CD32 antibody (ThermoFisher, 14-0161-82) for 10 minutes at 4°C and then incubated for 30 min at room temperature with primary antibodies including CD45-BV785 (BioLegend, 103149) CD11b-PE (BioLegend, 12-0112-82), CD68-FITC (BioLegend 137006), CD206-PE/Dazzle (BioLegend, 141732), and MHCII-APC/Cy7 (BioLegend, 107628). Following primary antibody incubation, cells were re-suspended in propidium iodide (PI, 2µL/mL) as a viability dye for 1 minute on ice (Life Technologies P3566). PI was then quenched with twice the volume of staining buffer and centrifuged at 450 x g for 1 minute. Flow cytometry analysis was performed using a Bio-Rad Ze5 Flow Cytometer and data was analyzed with FlowJo 10 software.

***Flow Cytometry Analysis of Acute Muscle Inflammation:*** At day 3 post-BaCl_2_ induced muscle injury both TA muscles from each mouse were pooled, finely minced, and digested for 60 min at 37°C for in Hank’s balanced salt solution lacking calcium and magnesium (HBSS^-/-^, Fisher Scientific), supplemented with 250 U/mL collagenase II (ThermoFisher), 4.0 U/mL dispase (Sigma-Aldrich), and 2.5 mmol/L CaCl_2_ (Sigma-Aldrich). The resulting digest solution was filtered through a 40-μm cell strainer and centrifuged at 350 x g for 5 min. Cells were Fc blocked with a CD16/CD32 antibody (ThermoFisher, 14-0161-82) for 10 minutes at 4°C and then incubated for 30 min on ice with primary antibodies including CD45-PE, Ly-6G-FITC (1A8) (BD Pharmingen, Franklin Lakes, NJ), CD64-APC (X54-5/7.1), CD11c-APCe780, (Biolegend, San Diego, CA), and Live/Dead fixable violet (ThermoFisher). Flow cytometry was performed using a LSRFortessa cell analyzer (BD Biosciences, San Jose, CA) and data analyzed with FlowJo 10 software.

***RNA extraction and cDNA synthesis:*** Muscle was homogenized in TRIzol reagent and RNA was isolated by Phenol/Chloroform extraction. RNA yield was determined using a NanoDrop Spectrophotometer (Nanodrop 2000c). Genomic DNA was removed by incubation with DNase I (Ambion, AM2222) followed by its heat inactivation. Total RNA (1 µg) was reversed transcribed to cDNA using SuperScript™ VILO™ Master Mix (Invitrogen, 11-755-050).

***LC-MS/MS based metabolipidomic profiling of muscle tissue:*** Muscle samples were mechanically homogenized in 1 mL of 50 mM phosphate, pH 7.4 with 0.9% saline (PBS) using a bead mill with reinforced tubes and zirconium beads (Precellys). The tissue homogenates were centrifuged at 3,000 x g for 5 min and the supernatant was collected. Supernatants (0.85 ml) were spiked with 5 ng each of 15(S)-HETE-d8, 14(15)-EpETrE-d8, Resolvin D2-d5, Leukotriene B4-d4, and Prostaglandin E1-d4 as internal standards (in 150 µl methanol) for recovery and quantitation and mixed thoroughly. The internal standard spiked samples were applied to conditioned C18 cartridges, washed with 15% methanol in water followed by hexane and then dried under vacuum. The cartridges were eluted with 2 x 0.5 ml methanol with 0.1% formic acid. The eluate was dried under a gentle stream of nitrogen. The residue was re-dissolved in 50 µl methanol-25 mM aqueous ammonium acetate (1:1) and subjected to LC-MS analysis.

HPLC was performed on a Prominence XR system (Shimadzu) using Luna C18 (3µ, 2.1x150 mm) column. The mobile phase consisted of a gradient between A: methanol-water-acetonitrile (10:85:5 v/v) and B: methanol-water-acetonitrile (90:5:5 v/v), both containing 0.1% ammonium acetate. The gradient program with respect to the composition of B was as follows: 0-1 min, 50%; 1-8 min, 50-80%; 8-15 min, 80-95%; and 15-17 min, 95%. The flow rate was 0.2 ml/min. The HPLC eluate was directly introduced to ESI source of QTRAP5500 mass analyzer (ABSCIEX) in the negative ion mode with following conditions: Curtain gas: 35 psi, GS1: 35 psi, GS2: 65 psi, Temperature: 600 ˚C, Ion Spray Voltage: -1500 V, Collision gas: low, Declustering Potential: -60 V, and Entrance Potential: -7 V. The eluate was monitored by Multiple Reaction Monitoring (MRM) method to detect unique molecular ion – daughter ion combinations for each of the lipid mediators using a scheduled MRM around the expected retention time for each compound. Optimized Collisional Energies (18 – 35 eV) and Collision Cell Exit Potentials (7 – 10 V) were used for each MRM transition. Spectra of each peak detected in the scheduled MRM were recorded using Enhanced Product Ion scan to confirm the structural identity. The data were collected using Analyst 1.7 software and the MRM transition chromatograms were quantitated by MultiQuant software (both from ABSCIEX). The internal standard signals in each chromatogram were used for normalization, recovery, as well as relative quantitation of each analyte.

LC-MS data was analyzed using MetaboAnalyst 4.0 (Chong et al., 2018). Analytes with >50% missing values were removed from the data set and remaining missing values were replaced with half of the minimum positive value in the original data set. Heat maps were generated in MetaboAnalyst 4.0 using the Euclidean distance measure and Ward clustering algorithm following autoscaling of features (analytes) without data transformation. Unsupervised principle component analysis (PCA) and volcano plots were generated in R using the FactoMineR/factoextra and EnhancedVolcano packages respectively. Ellipse.type="confidence" and "ellipse.level = 0.95" were used on PCA score plots. Targeted statistical analysis by two-way ANOVA was performed on pooled analyte concentrations from major lipid mediator biosynthetic pathways (Figure 3B) as well as pre-selected individual analytes of interest (Supplemental Figure 2).

***Muscle Force Testing:*** Mice were anesthetized with 2% isoflurane and placed on a heated platform. The distal half of the TA muscle was isolated by dissecting the overlying skin and fascia. The knee joint was immobilized and a 4–0 silk suture tied around the distal TA tendon which was severed from its boney insertion and tied to the lever arm of a servomotor (6650LR, Cambridge Technology). A saline drip warmed to 37°C was continuously applied to the exposed muscle. The peroneal nerve was stimulated with 0.2 ms pulses using platinum electrodes with the stimulation voltage and muscle length adjusted to obtain optimal muscle length (L_o_) maximum isometric twitch force (P_t_). The TA was then stimulated at increasing frequencies while held at L_o_ until maximum isometric tetanic force (P_o_) was achieved. One-minute rest was allowed between each tetanic contraction. Muscle length was then measured with calipers and optimum fiber length (L_f_) determined by multiplying L_o_ by the TA muscle L_f_/L_o_ ratio of 0.6 (Burkholder, Fingado, Baron, & Lieber, 1994). The cross-sectional area (CSA) of the muscle was calculated by dividing muscle mass by the product of L_f_ and 1.06 mg/mm^3^ (Mendez and Keys, 1960) (Mendez & Keys, 1960). Specific P_o_ (sP_o_) was calculated by dividing P_o_ by muscle CSA.

***Bone Marrow-Derived Macrophage Culture:*** Bone marrow was collected from tibias and femurs of young (4-6 mo) or aged (26-28 mo) female C57BL/6 mice and cultured for 7 days at 37°C and 5% CO_2_ in DMEM (Gibco, 11995-073) supplemented with 10% FBS, antibiotics, and 20 ng/mL recombinant murine GM-CSF (Bio-legend, 576304). Cells were washed with PBS to remove non-adherent cells and adherent bone marrow-derived macrophages (BMMs) were detached by incubation in TrypLE Select at 37°C (Gibco, 12563011) followed by gentle cell scraping. BMMs were plated into 96 well plates at a density of 1 × 10^5^ cells/well in growth media lacking GM-CSF and allowed to adhere overnight before use. MΦ were pre-treated for 15 min with RvD1 (1-100 nM) or vehicle (0.1% ethanol) in growth media which was then replaced with pHrodo Green *E. Coli* Bio Particles (Invitrogen, P35366) prepared in hanks balanced salt solution containing calcium and magnesium (HBSS^+/+^) and incubated at 37°C for 1 hour. Non-engulfed *E. coli* BioParticles were removed by washing with HBSS^+/+^ and intracellular fluorescence measured using a plate reader at an excitation/emission of 509/533 nm.

**References**

Burkholder, T. J., Fingado, B., Baron, S., & Lieber, R. L. (1994). Relationship between muscle fiber types and sizes and muscle architectural properties in the mouse hindlimb. *J Morphol, 221*(2), 177-190. doi:10.1002/jmor.1052210207

Chong, J., Soufan, O., Li, C., Caraus, I., Li, S., Bourque, G., . . . Xia, J. (2018). MetaboAnalyst 4.0: towards more transparent and integrative metabolomics analysis. *Nucleic Acids Res, 46*(W1), W486-W494. doi:10.1093/nar/gky310

Mendez, J., & Keys, A. (1960). Density and Composition of Mammalian Muscle. *Metabolism, 9*, 184-188.
